# The Impact of the Secondary Binding Pocket on the Pharmacology of Class A GPCRs

**DOI:** 10.3389/fphar.2022.847788

**Published:** 2022-03-09

**Authors:** Attila Egyed, Dóra Judit Kiss, György M. Keserű

**Affiliations:** Medicinal Chemistry Research Group, Research Centre for Natural Sciences, Budapest, Hungary

**Keywords:** GPCR (G-protein coupled receptor), allosteric, bitopic, selectivity, functional selectivity

## Abstract

G-protein coupled receptors (GPCRs) are considered important therapeutic targets due to their pathophysiological significance and pharmacological relevance. Class A receptors represent the largest group of GPCRs that gives the highest number of validated drug targets. Endogenous ligands bind to the orthosteric binding pocket (OBP) embedded in the intrahelical space of the receptor. During the last 10 years, however, it has been turned out that in many receptors there is secondary binding pocket (SBP) located in the extracellular vestibule that is much less conserved. In some cases, it serves as a stable allosteric site harbouring allosteric ligands that modulate the pharmacology of orthosteric binders. In other cases it is used by bitopic compounds occupying both the OBP and SBP. In these terms, SBP binding moieties might influence the pharmacology of the bitopic ligands. Together with others, our research group showed that SBP binders contribute significantly to the affinity, selectivity, functional activity, functional selectivity and binding kinetics of bitopic ligands. Based on these observations we developed a structure-based protocol for designing bitopic compounds with desired pharmacological profile.

## Introduction

**FIGURE 1:**
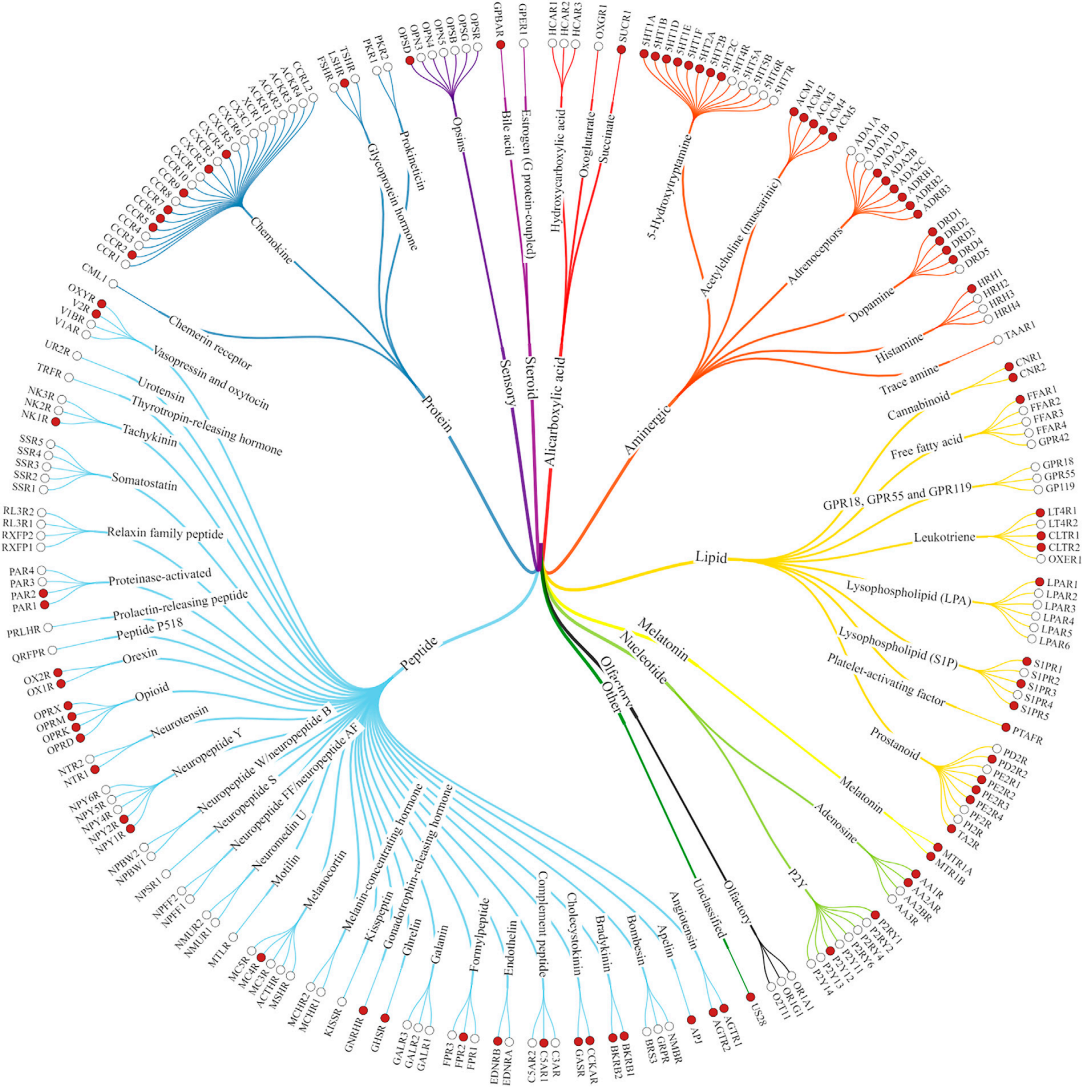
Class A GPCRs. The structures of the receptors marked with red dots have already been solved experimentally (Kooistra et al., 2021).

G-protein coupled receptors (Figure 1) are among the most popular targets for drug discovery and the development of novel therapeutic and pharmacological tools. One third of the drugs currently approved by the Food and Drug Administration affects one of the GPCRs (Sriram and Insel, 2018). They are critical in signal transduction of hormones and neurotransmitters, and consequently are pharmacological targets for many diseases (Overington et al., 2006). Furthermore, studying these receptors may help to elucidate the signaling mechanisms in cells, as they play a crucial role in the regulation of both central and peripherial neurological and physiological processes. Detailed understanding of these processes facilitates the development of more targeted therapies (Christopoulos, 2014).

**FIGURE 2:**
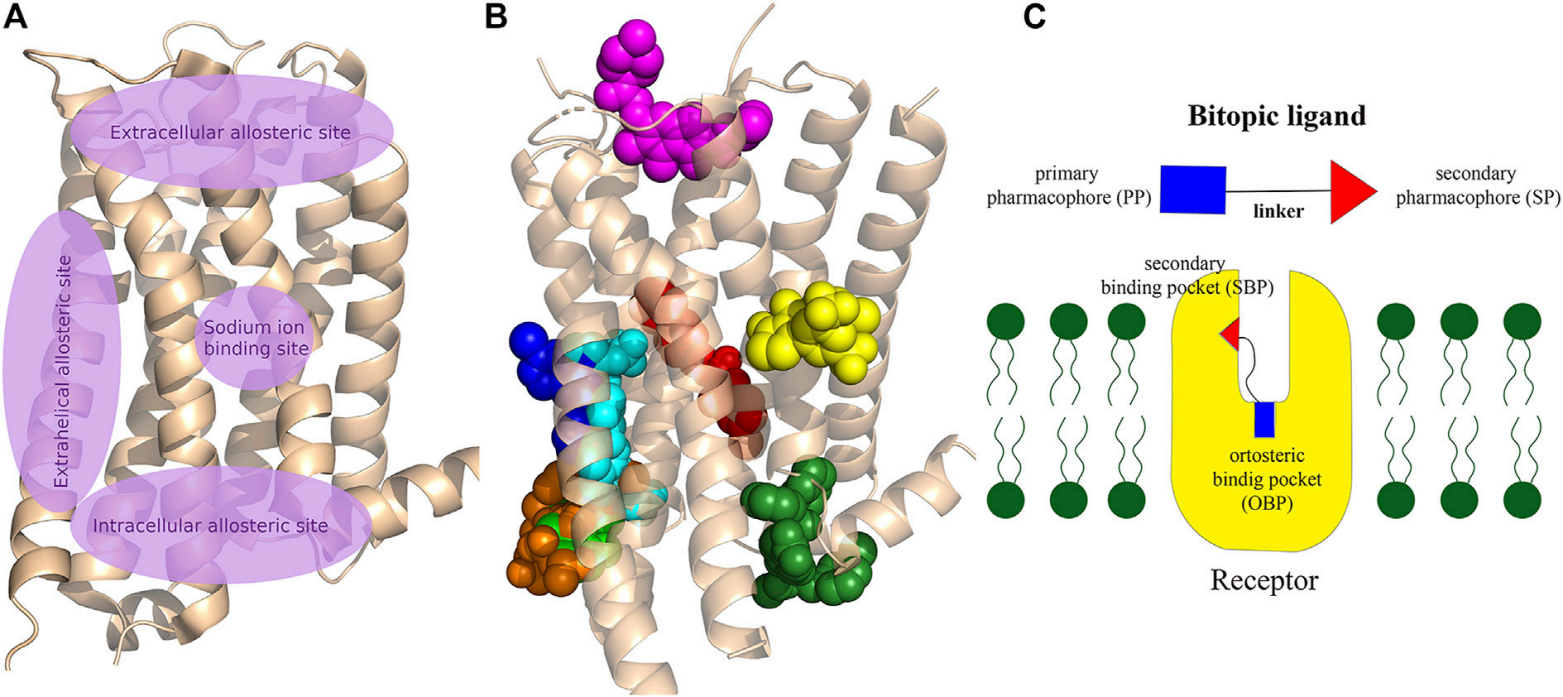
**(A)** Schematic representation of the main allosteric sites in Class A GPCRs. The OBP, where the endogenous ligands bind to the receptor, is located between the extracellular allosteric site and the sodium binding site, deep in the crevice of the receptor formed by the transmembrane helixes. Some allosteric sites are clearly separated from OBP, while others can be considered as an expansion of the orthosteric pocket. **(B)** Visualisation of allosteric binding sites for some important compounds related to the review: mevidalen in the D_1_R (green, PDB code: 7LJD), AP8 in FFAR1 (cyan, PDB code: 5TZY), ORG27569 in CB_1_ (red, PDB code:6KQI), MIPS521 in A_1_R (yellow, PDB code: 7LD3), LY2119620 in M_2_R (magenta, PDB code:4MQT), Cmpd-15PA in β_2_AR (dark green, PDB code: 5X7D), AS408 in β_2_AR (dark blue, PDB code:6OBA), cmpd-6fa in β_2_AR (orange, PDB code: 8N48). Cholesterol was shown to bind to extrahelical binding sites to different TMs that could not be depicted on the figure to maintain clarity. For details please see the recent review of Jakubík and El-Fakahany (2021) and for a review of the allosteric sites at the receptor–lipid bilayer interface please see Wang et al. (2021) **(C)** Schematic structure of a bitopic compound. The primary pharmacophore that binds to the OBP is linked through a linker to the secondary pharmacophore binding to the SBP.

GPCRs have multiple ligand binding sites, the orthosteric binding pocket and a generally separated less conserved allosteric secondary binding pocket (Christopoulos, 2014). Basically, the endogenous ligand binds to the OBP. SBPs are found in both the extracellular and intracellular parts of the receptor (Figure 2), some of these binding sites are well separated from the OBP while others may have extended binding pocket-like features such as the 5-HT_2A_ aripirazole structure (PDB: 7VOE) (Chen et al., 2021).

These secondary binding sites have become key to achieve the right subtype selectivity and functionality. Therefore, a lot of effort was given to the research of allosteric binding sites and allosteric modulators. A large number of allosteric modulators of GPCRs that bind to the extracellular or intracellular domains were identified. The combination of a primary pharmacophore (PP) binding to the OBP and a secondary pharmacophore (SP) binding to the SBP resulted in bitopic compounds (Figure 2C) that combine the pharmacological properties of both types of ligands defining a new unique pharmacological profile. One of the first published bitopic molecules of this type is methoctramine that acts as an antagonist at the muscarinic receptor M_2_R (Melchiorre et al., 1987).

In this review we would like to give only a brief insight into class A GPCR structures and the world of allosteric modulators as several reviews have been published in the field. Mainly, we discuss in detail the recent advances in bitopic ligands, while we close the review with an outlook towards the design approaches in the field.

## Ligand Binding Pocket Revealed by Experimental Structures

**FIGURE 3:**
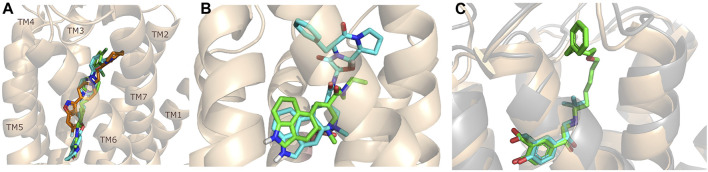
Structures of some important bitopic compounds. **(A)** The unusual “upside-down” binding mode of cariprazine (green) and aripiprazole (cyan) in the inactive 5-HT_2A_ structure. Risperidone (orange) is shown as a reference to highlight the cryptic pocket opened up by aripiprazole and cariprazine. **(B)** The aligned LSD (Wacker et al., 2017) and ergotamine (Wacker et al., 2013) 5-HT_2B_ structure highlighting that the introduction of an SP can influence the binding mode of the PP. The figure was reproduced from Supplementary Figure S7 of our paper (Egyed, A et al. Controlling Receptor Function from the Extracellular Vestibule of G-Protein Coupled Receptors. Chem. Commun. 2020, 56 (91), 14167–14170) (Egyed et al., 2020). **(C)** The binding mode of salbutamol (cyan) and salmeterol (green) (Masureel et al., 2018) in the β_2_R highlighting the important role of ECL2 as discussed in more detail in the binding kinetic section of this review.

Recent advances in X-ray crystallography and cryo electron microscopy provided many new structures of GPCRs complexed with allosteric ligands. As of early December 2021, 57 GPCR structures containing allosteric ligands have been found in GPCRdb (Kooistra et al., 2021), these structures cover 20 receptor types and three different states; active, inactive and intermediate. Among allosteric ligands, examples of positive (PAM) and negative allosteric modulators (NAM) can be found. The collection of the published GPCR structures with allosteric ligands is available in the supporting information (Kooistra et al., 2021) (Supplementary Table S1). In addition, a significant number of active structures have become accessible, which may provide more information on the mechanism of receptor activation and offer considerable support for drug design, although few of these are allosteric ligands. Among them, 35 active aminergic GPCR structures have been published in the last 2 years (Supplementary Table S2) (Kooistra et al., 2021). These include 7 serotonin (Kim et al., 2020; Peiyu Xu et al., 2021a; Huang et al., 2021) (5-HTR), 15 dopamine (Zhuang et al., 2021a; Xiao et al., 2021; Zhuang et al., 2021b; Yin et al., 2020; Peiyu Xu et al., 2021b) (DR), 1 histamine (Xia et al., 2021) (HR), 1 muscarinic (Staus et al., 2020) (MR) and 11 adrenergic (Lee et al., 2020; Fan Yang et al., 2021; Yuan et al., 2020; Su et al., 2020; Xinyu Xu et al., 2021; Zhang et al., 2020; Nagiri et al., 2021) (AR) receptor structures. Out of these complexes, 20 structures contain allosteric modulators but not obviously in the SBP, while 10 were co-crystallized with bitopic ligands bound both the OBP and the SBP. The discussion of the structures in detail is out of scope of this review, however we highlight here the new cariprazine and aripiprazole bound 5-HT_2A_ structures (Figure 3A). (Chen et al., 2021) Interestingly, both compounds display an unexpected binding mode with their secondary binding motif exploring a binding pocket deep in the receptor instead of engaging with the extracellular secondary binding pocket. In the dopamine D_2_ and D_3_ receptors (D_2_R, D_3_R) the docking positions of aripiprazole so far have shown that 4-(2,3-dichlorophenyl)piperazine PP is located roughly parallel to the membrane plane and close to S5.42 and F6.51. The dihydroquinoline secondary pharmacophore is located at the junction of transmembrane helices (TM) 1, TM2, TM7 or TM3, TM5 and extracellular loop (ECL) 2. However, in the 5-HT_2A_ crystal structures of aripiprazole and cariprazine the ligands are located in an “upside-down” binding mode. The 2,3-dichlorophenyl PP occupies the orthosteric site and faces the extracellular region, but the dihydroquinoline SP vertically penetrates the hydrophobic pocket formed between TM5 and TM6 and interacts with residues L247^5.51^, V333^6.45^ and C337^6.49^ and forms π-π interactions with residues F332^6.44^ and W338^6.48^. Upon binding of aripiprazole, a conformational rearrangement occurs resulting in an increase in the size of the binding pocket. Induced docking with D_2_R was used to reproduce the “upside-down” binding pose of aripiprazole and cariprazine. Compared with the rigid docking, a much lower binding energy was calculated in the induced-fit docking, indicating that the upside-down binding mode represents a more stable conformation of D_2_R (Chen et al., 2021).

## Allosteric Modulators in the Class A GPCR Field

Allosteric binding sites (Figures 2A,B **)** have attracted increasing interest in order to develop more selective agents with fewer side effects (Congreve et al., 2017a; Chan et al., 2019). Allosteric sites are typically less conserved than orthosteric pockets and therefore they could provide greater selectivity and better control over the dynamical equilibrium of the receptor. Following the classic structural architecture of a class A GPCR, the orthosteric binding pocket is formed by the transmembrane helixes while the extracellular loops and the N-terminus of the peptide chain define the secondary binding domain. It should be mentioned, however, that there are other allosteric sites (e.g., extrahelical sites at the protein-membrane interface, intracellular sites at the signalling domain or intrahelical sodium site) available. Allosteric ligands can modify the biological response, they can stabilise the active or inactive conformation that is potentially linked to biased signalling or partial agonism (Wakefield et al., 2019). Based on spectroscopic and structural studies, conformational changes in the receptor govern the activation of signalling pathways. Characterization of interactions with intracellular partners guiding the allosteric process is a major challenge and can only be fully understood by using a combination of different methodologies (Liu et al., 2012; Masureel et al., 2018; Frei et al., 2020). Most allosteric modulators have been discovered serendipitously by high throughput screening (HTS) campaigns (Bian et al., 2020). Due to the vastness of the topic and the number of reviews published in the last years, we will only provide a brief insight into the world of allosteric modulators.

**FIGURE 4:**
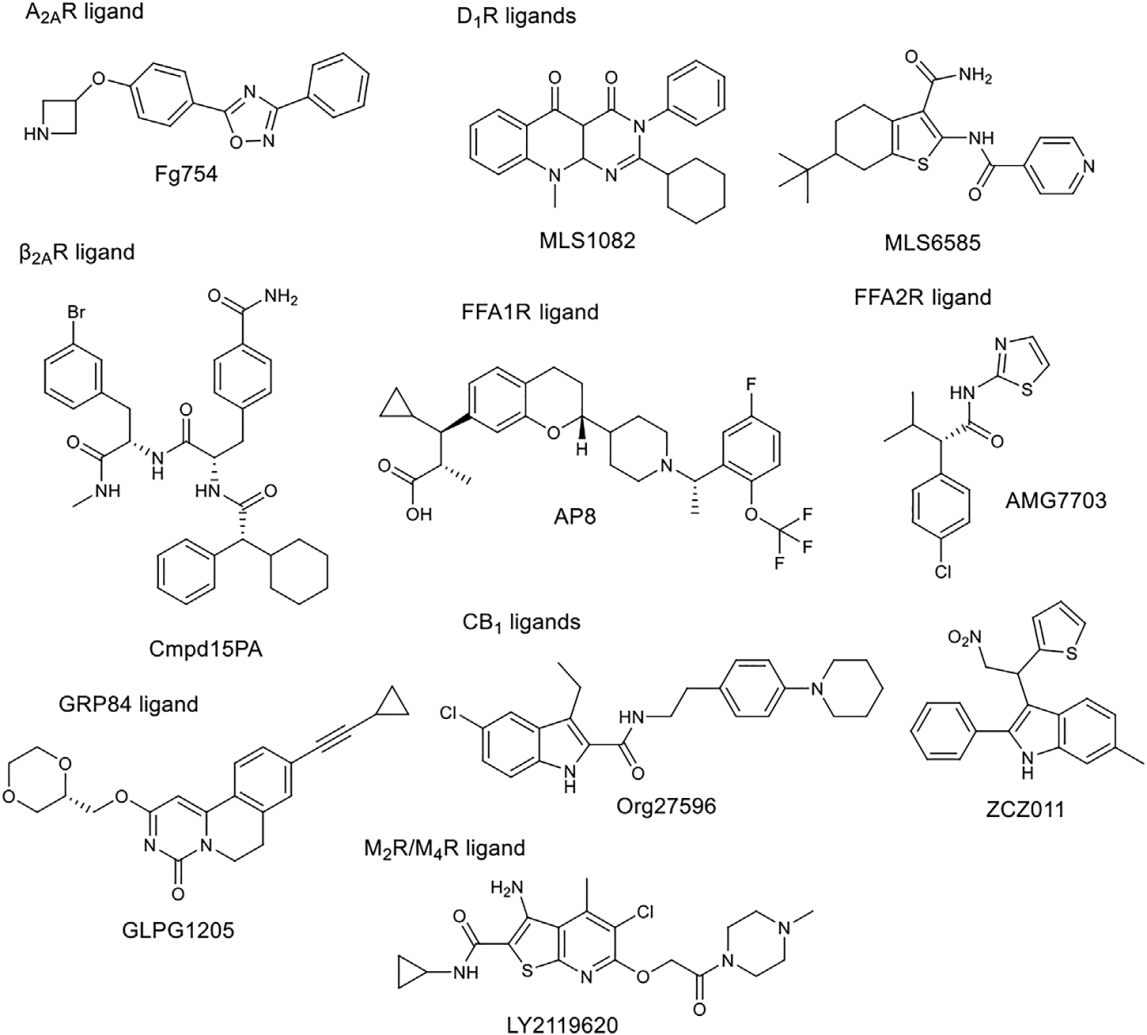
Chemical structure of selected allosteric modulators.

The tissue distribution and relative expression of the four adenosine receptor (AR) subtypes A_1_R, A_2A_R, A_2B_R and A_3_R regulate the physiological effects of endogenous adenosine. Adenosine receptors are expressed in most tissues and major organs, including brain, heart, kidney, skin, adipose tissue, immune cells, lung and liver. The four adenosine receptor subtypes can be broadly classified into two classes. Baressi et al. described a type of A_2B_R allosteric modulators with good selectivity over the other subtypes, these compounds contain a 1,3-substituted indole unit (Barresi et al., 2021a; Barresi et al., 2021b). Lu et al. established a fragment screening method using mass spectrometry to screen GPCR ligands, identifying an A_2A_R NAM. Fg754 (Figure 4) contains a specific acetidine moiety that forms bonds in the sodium ion pocket. Based on molecular dynamics (MD) simulations, it may overlap with the orthosteric binding site, probably acting in a mixed mode. The compound could thus be a new starting point for the development of allosteric modulators or bitopic compounds (Yan Lu et al., 2021). The A_1_R and A_3_R preferentially bind to G_i/o_ proteins to inhibit adenylate cyclase activity, while the A_2A_R and A_2B_R preferentially bind to G_s_ proteins to stimulate adenylate cyclase activity. Like other GPCRs, adenosine receptors can interact with different G-protein subtypes. In addition, A_2B_R has been suggested to couple to both G_i/o_ and G_q_ proteins (Linden et al., 1999; Gao et al., 2018), while A_1_R has been shown to couple to both G_s_ and G_q_ proteins (Cordeaux et al., 2004). In addition to G_α_ signalling, G_β_γ dimers released following G protein activation can interact with effector proteins to modulate intracellular signalling. Beside G-protein-dependent signalling, adenosine receptors can also signal through G-protein-independent effectors. One of the best described G-protein-independent pathway is initiated following recruitment of arrestin adaptor proteins (β-arrestin1 and β-arrestin2). This process is typically preceded by G-protein coupled receptor kinase mediated phosphorylation, but recent studies have shown the possibility of phosphorylation independent β-arrestin recruitment for several GPCRs, including A_3_R. Arrestin recruitment has been investigated primarily in A_2B_R and A_3_R and there is limited evidence that A_1_R or A_2A_R can recruit β-arrestin. McNeill et al. have discussed in detail the effects of allosteric modulators belonging to different subtypes on distorted signalling, which will not be discussed in detail below (McNeill et al., 2021).

Free fatty acids may act as signalling molecules at FFA receptors (FFARs). Free fatty acids of different chain lengths and saturation states activate FFARs as endogenous agonists by binding at the orthosteric receptor site. Following FFAR deorphanisation, a number of ligands targeting allosteric sites on FFARs have been identified with the aim of developing drugs for metabolic, (auto)inflammatory, infectious, endocrine, cardiovascular and renal diseases. In 2021, Grundmann et al. published a detailed review (Grundmann et al., 2021) on free fatty acid receptors, describing in detail the subtypes (FFAR1, FFAR2, FFAR3, FFAR4, GPR84), their function, structures and outlined the importance and challenges of allosteric modulators. FFAR1 is the most studied subtype. Although the biology of the receptors is still largely elusive, a large body of research evidence has accumulated around ligand-receptor interactions and their associated signalling capabilities. At least three distinct groups of FFAR1-activating ligands can be distinguished: 1) endogenous/orthosteric agonists (long-chain fatty acids), partial allosteric agonists (fasiglifam, MK-8666, AM 837), and full allosteric agonists (AM 1638, AP8) (Figure 2B, Figure 4). These groups differ not only in their apparent binding sites (Figure 2B) on the receptor, but also in their ability to induce different downstream signalling pathways of FFAR1, ultimately leading to different results in the phenotype of the FFA1 receptor *in vivo*. New results on allosteric FFAR2 ligands (AMG 7703, AZ1729, Compound 58) (Figure 4), show promising pharmacological properties and have generated new interest in this target, considering new allosteric modalities. GLPG1205 (Figure 4), an antagonist and negative allosteric modulator of GPR84, showed promising preclinical results in models of idiopathic pulmonary fibrosis, but was later discontinued from development. Allosteric targeting of small-, medium-, and long-chain fatty acid receptors is a promising approach to address a variety of therapeutic areas, demonstrating the biological diversity and drug target attractiveness of members of this receptor family (Grundmann et al., 2021).

The cannabinoid receptor type 1 (CB_1_) was first discovered as the main target for Δ9-tetrahydrocannabinol (THC), the psychoactive compound in Cannabis. CB_1_ was first identified in rat and later cloned from a human brain cDNA library. Widely known CB_1_ agonists are synthetic cannabinoids and THC analogues, such as HU-210 (Howlett et al., 1990), CP55940 (Kapur et al., 2009), and WIN55212 (Felder et al., 1995). The CB_1_ receptor preferentially binds a G_i_ protein and its activation leads to a decrease in cyclic adenosine monophosphate (cAMP) levels in cells. Other signalling pathways have also been investigated, focusing primarily on ERK1/2 phosphorylation. ERK signalling is hypothesised to play a role in cocaine addiction, and together with cAMP, to be an important regulator of synaptic plasticity, memory and learning. Inhibition of CB_1_ proved effective in the treatment of obesity with antagonists or inverse agonists, but they were later withdrawn from the market due to adverse psychiatric side effects (anxiety, suicidal ideation). Several new strategies to avoid potential side effects have been analysed, one of them being the development of allosteric modulators. Leo and Abood reviewed the physiological and pathophysiological roles of CB_1_, described the signalling mechanisms, and investigated CB_1_ biased signaling (Leo and Abood, 2021). Based on agonist-bound solvated molecular structures and biased allosteric modulators they look at possible molecular mechanisms of CB_1_ signalling. Mielnk et al. present the *in vitro* and *in vivo* profiles of several NAMs (Org27569, PSNCBAM-1, ABM300, Pepcan-12, Pregnenolone, and cannabidiol) and PAMs (ZCZ011, GAT211, Lipoxin A4, LDK1258) in detail (Figure 2B, Figure 4). They concluded that CB_1_ PAMs in anxiety and depression while CB_1_ NAMs—in combination with cannabidiol—in psychosis could be promising (Mielnik et al., 2021).

Che and Roth have provided a detailed summary of the pharmacology, ligands (orthosteric, allosteric), and structures of opioid receptors (OR) (Che and Roth, 2021). Activating µ-opioid receptor (MOR) causes serious side effects, which are the root of the current opioid crisis. In their review, potential strategies and targets for developing opioid alternatives were discussed. Separately, they list OR biased agonists, allosteric modulators, multitarget ligands and peripherally restricted ligands. The complexity of signalling pathways should be considered in the therapeutic potential of biased agonists, and allosteric modulators are alternative means to modulate more precisely the action of endogenous or exogenous ligands. As opioid receptors are widely expressed in the peripheral system, the use of ligands restricted to this system would avoid central nervous system induced side effects. Simultaneous targeting of multiple opioid and non-opioid receptors may result in safer analgesics (Che and Roth, 2021).

The family of aminergic GPCRs includes adrenergic, dopamine, serotonin, histamine, muscarinic and trace amine receptors. These receptors have several similarities, they bind monoamine neurotransmitters, acetylcholine, or trace amines. They share common features in sequence, structure and function. Ergotamine (Figure 3B) can bind to 22 aminergic receptors with K_i_ values less than 1 µM (Peng et al., 2018). Other examples can be found in the literature, such as chlorpromazine, clozapine, thioridazine, olanzapine which have good affinity for several aminergic GPCRs (Roth et al., 2004). On the other hand, it would be important to produce drugs that have subtype and functional selectivity to avoid side effects.

In the field of adrenergic receptors, Wu and co-workers have discussed in detail the binding of endogenous ligands to different receptors, the mechanism of β-adrenergic and α_2_ receptor attenuation, distorted signal transduction, subtype selectivity, and selectivity between the main types. Insights into the allosteric modulation of β_2A_R were provided. They also reported on the results obtained with different modalities. The cholesterol binding site was recently described in detail by Sarkar and Chattopadhyay (2020) The arrangement of the 7 TMs in each class of GPCRs results in a groove at the lipid interface formed by TM3/4/5, and in β_2A_R, to this site the binding of PAMs and NAMs were identified. GPCRs use the cytoplasmic surface to interact with intracellular partners with small molecules binding at this site discovered primarily in chemokine receptors. Only Cmpd15PA (Figure 2B, Figure 4) in β_2A_R targets this site outside the chemokine subfamily. These small molecules are all NAMs. Cmpd15PA has little interaction with the G protein, but stabilizes the receptor inactive state through extensive interactions with TM1, TM2, TM6, TM7, H8 and intracellular loop 1 (Wu et al., 2021).

The five dopamine receptor subtypes (D1–5) are activated by the endogenous catecholamine dopamine. The D1-like family comprises dopamine D_1_ and D_5_ receptors that mainly couple to the G_s_ G-protein and thereby stimulate cAMP production. The D_2_-like family includes D_2_, D_3_, and D_4_ receptors, that couple to G_i/o_ G-proteins and attenuate cAMP production (British Pharmacological Society, 2021). Fasciani et al. have presented allosteric modulators of the DR, the bitopic compound SB269652 has been analysed in detail. Mao et al. describe the role of different dopamine receptor allosteric modulators in the treatment of Parkinson’s disease. DR allosteric modulators represent an alternative and promising strategy for drug discovery of GPCRs with high selectivity and low side effects (Mao et al., 2020). Like many other receptors, the classical approach to D_1_R is the development of orthosteric ligands, but this has several drawbacks from a therapeutic point of view. D_1_R agonists have narrow therapeutic window, can induce seizures and hypotensive side effect. PAMs are a more useful approach because they potentiate the effect of endogenic dopamine, the available dopmaine level provides a natural ceiling effect for PAM activity, and endogenous spatial and temporal regulation of dopamine-mediated stimulation is maintained. To date, seven D_1_R PAM structural classes have been discovered. Two of these (MLS1082 and MLS6585) were discovered in 2018 by Luderman and colleagues using HTS (Luderman et al., 2018) (Figure 4). Subsequently, MLS1082 was investigated in a SAR study and they identified several analogues that enhanced dopamine-induced D_1_R activation (Luderman et al., 2021).

There are five subtypes of the muscarinic acetylcholine receptor. The different subtypes show high degree of homology in the transmembrane domains. In recent years, the structures of all five have been resolved by X-ray crystallography (Vuckovic et al., 2019; Thal et al., 2016; Kruse et al., 2013; Kruse et al., 2012; Haga et al., 2012). In a review, Jakunik and El-Fakahany provide a detailed analysis of allosteric adhesion, the molecular mechanisms of action, and present specific modulators. The diversity of the effects of allosteric modulators and the studies on them will greatly influence the development of new therapies. Selective PAMs (LY2119620) (Figure 2B, Figure 4), which have therapeutic potential in the treatment of Alzheimer’s disease or schizophrenia, show encouraging results (Conn et al., 2009; Bock et al., 2018; Jakubik and El-Fakahany, 2020).

Biochemically, 5-hydroxytryptamine (5-HT) is derived from the amino acid tryptophan, undergoing hydroxylation and decarboxylation processes that are catalyzed by tryptophan hydroxylase and aromatic L-amino acid decarboxylase, respectively. As a biogenic amine, 5-HT plays important roles in cardiovascular function, bowel motility, platelet aggregation, hormone release and psychiatric disorders. 5-HT achieves its physiological functions by targeting various 5-HT receptors (5-HTRs), which are composed of six classes (5-HT_1_, 5-HT_2_, 5-HT_4_, 5-HT_5_, 5-HT_6_, and 5-HT_7_ receptors, a total of 13 subtypes) and a class of cation-selective ligand-gated ion channels, the 5-HT_3_ receptor. Barnes et al. have published a review (Barnes et al., 2021) detailing each subtype, describing their functions and pharmacology one by one and discuss known allosteric ligands. They find that 5-HT receptors are less involved in allosteric modulation than other GPCRs (e.g., muscarinic, GABA), with the possible exception of 5-HT_3_R. However, from some structures with ergoline, it becomes clear that, in addition to the classical OBP, some 5-HT receptors have an extended binding site very similar to that described for muscarinic allosteric ligands. Such molecular targets may offer attractive strategies for new therapies (Barnes et al., 2021).

## Bitopic Ligands to Study Selectivity and Functional Selectivity of Class A GPCRs

As outlined in the introduction, our primary focus is on bitopic compounds in this review. These compounds combine the efficiency of orthosteric ligands and the diversity of allosteric SPs by interacting with both binding sites simultaneously. This gives bitopic ligands an advantage over allosteric modulators, as the latter need an orthosteric ligand to exert their effect. This may be important in cases where endogenous substrate depletion contributes to the pathogenesis of disease, such as in Parkinson’s and Alzheimer’s diseases, but there are further examples in metabolic disorders. The key strucutural moieties of bitopic compounds (PP, SP and linker, depicted on Figure 3C) have different roles. PP is classically considered to be responsible for functionality while SP can modulate binding affinity, selectivity as well as functional character and efficacy. The linker connects the two pharmacophores and may be responsible for the optimal binding poses by positioning the pharmacophores and affecting the pharmacology profile (Bethany et al., 2019).

In the design of bitopic compounds, the desired orthosteric binding motif should have high affinity for the selected receptor and ideally, the SP should provide high subtype selectivity while maintaining or even increasing affinity. In the case of a linker, the choice of attachment points and length must be appropriate, and the linker must be moderately flexible to allow the pharmacophores to bind properly. For agonists, it is important that the linker does not interfere with conformational changes induced by receptor activation (Valant et al., 2012; Lane et al., 2013; Fronik et al., 2017; Bethany et al., 2019). Reinecke et al. published a review on bitopic compounds in 2019, summarizing the new bitopic compounds that have been published in the last 5 years (Bethany et al., 2019). Here we therefore focus on compounds published in 2020–21, with a contextual analysis of previously published compounds where appropriate. In the following subsections, we discuss subtype selectivity and functional selectivity results separately.

### Receptor and Subtype Selectivity

Receptor and subtype selectivity is an important criterion for minimizing side effects, therefore tremendous efforts go into the development of compounds with designed binding profile.

**FIGURE 5:**
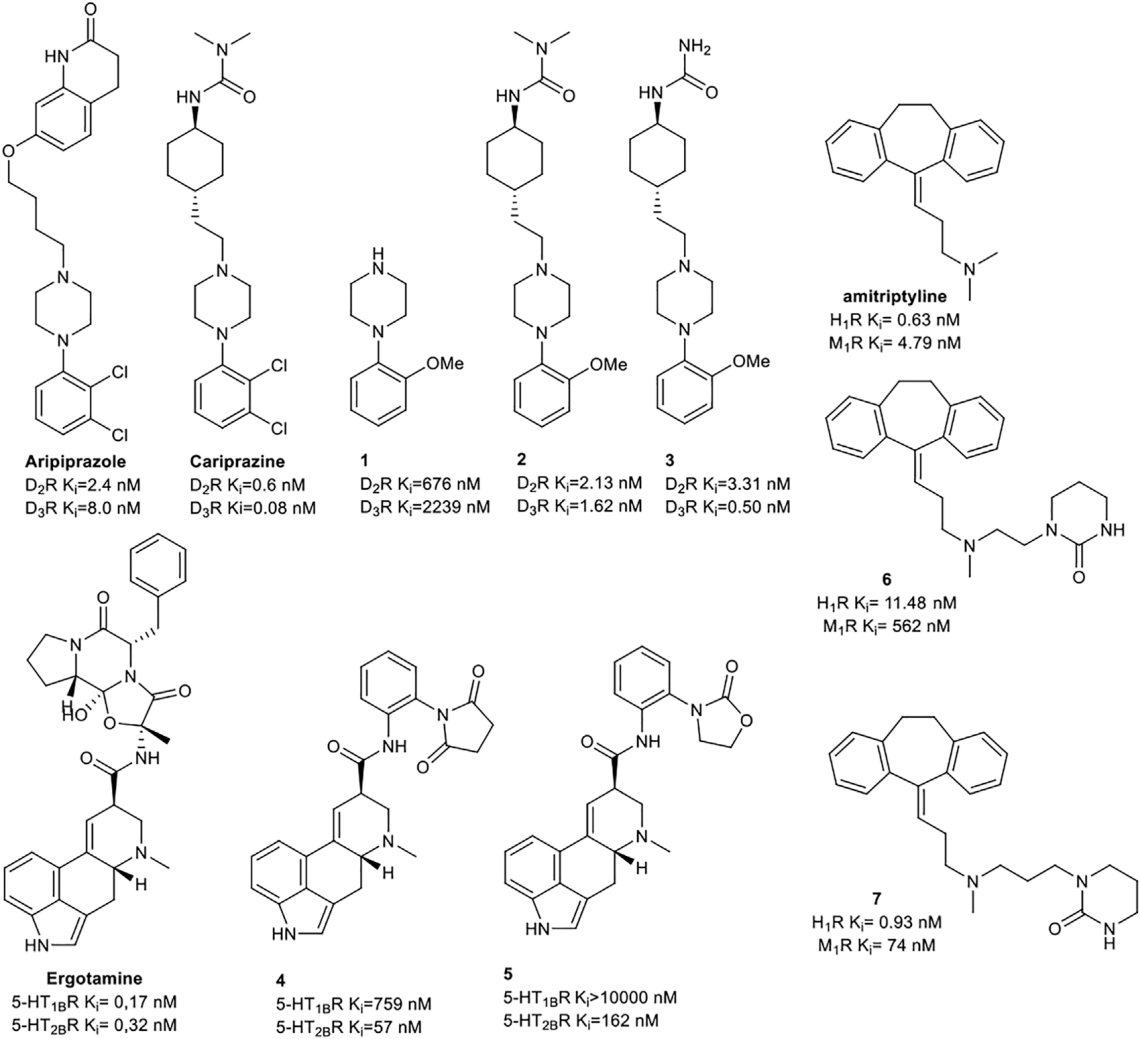
Designed bitopic ligands and the reference compounds in the study of Keserű et al. (Egyed et al., 2021).

Keserű et al. have developed a fragment based docking protocol to design specific receptor ligands. Based on the docking results, they have synthesized several compounds and demonstrated the usefulness of the method for the designing D_2_/D_3_, 5-HT_1B_/5-HT_2B_ and H_1_/M_1_ receptor ligands with improved selectivity (Figure 5). In the first two cases, the selectivity of the PP was reversed using the SP moiety, while in the third case, a selective compound was designed and synthesized for a receptor pair with very similar PP (Egyed et al., 2021).

The importance of bitopic compounds in the inhibition of dopamine receptors is demonstrated by second and third generation antipsychotics, including aripiprazole (Burris et al., 2002) and cariprazine (Ágai-Csongor et al., 2012). 2,3-dichlorophenyl-piperazine, that serves as PP in these compounds was changed to 2-methoxyphenylpiperazine (**1**) PP. Although this PP exhibits weak D_2_R selectivity, combined with a suitable SP group (**2,3**) its profile has been changed to mild D_3_R selectivity. The efficacy of this methodology was further tested on serotonin receptors. The LSD-like PP of ergotamine (Figure 3B) did not show subtype selectivity at the two selected serotonin receptors, but the designed compounds (**4,5)** with the modified SP already had significantly higher affinity at 5-HT_2B_R over 5-HT_1B_R. Although ergotamine was more potent, compounds **4** and **5** had much greater selectivity over it. Among the first-generation antihistamines, muscarinic acetylcholine M_1_ activity was a major problem due to side effects. Therefore, huge efforts were dedicated to the development of compounds with significant H_1_R receptor selectivity. Starting from amitriptyline having only 7-fold selectivity, bitopic compounds (**6**,**7**) were designed that demonstrated 50–80 fold selectivity over M_1_R (Egyed et al., 2021). The proposed protocol detailed in the design section of this review may be applied to other targets to achieve designed selectivity with bitopic compounds.

Tan et al. have exploited the basic 2-phenylcyclopropylmethylamine (PCPMA) scaffold (**8, 9**), whose analogues are known 5-HT_2C_R agonists (Cheng et al., 2015; Cheng et al., 2016a; Cheng et al., 2016b; Zhang et al., 2017), to design new bitopic compounds (Tan et al., 2020) (Supplementary Table S3). Here we discuss only a subset of these compounds. As secondary pharmacophore, 1,2,4-triazolylthiol ethers were used and a propyl chain was employed as a linker. The introduction of SP alone improved D_3_R activity 3-fold. A major leap forward was the realization that the alkyl side chain introduced on the amino group of PCPMA significantly improves subtype selectivity and D_3_R affinity. Next, they investigated the substituents of the aromatic ring of PCPMA. First, the ortho positioned 2-fluoroethoxy group was changed, whereby methoxy was found to be the optimal one, thus significantly improving the D_3_R affinity. The replacement of the fluorine atom by chlorine resulted in a moderate selectivity towards D_2_R, D_4_R, 5-HT_2C_R and a strong selectivity towards D_1_R and D_5_R (**10–12**). As these results could only approximate the values of the reference compound **13** (BP-897) (Supplementary Table S3) the strategy was changed and a buthylene linker was used instead of the propylene group, the SP was replaced by other aromatic rings (naphthyl, indolyl, and 4-pyridylphenyl) and an amide bond between the linker and the SP was introduced instead of thioether (**14–20**). For these compounds, only N-alkyl substituted variants have been prepared and the effects of several PPs have been investigated. When examining the racemic compounds, the compound containing 4-pyridylphenyl SP and dichlorophenyl PP (**20**) has more than 1000-fold selectivity towards the other DRs, with milder but still significant selectivity in the range of **17**, **18,** and **19** (Tan et al., 2020) (Table 1).

**TABLE 1:** Selected compounds from DR related selectivity studies (Battiti et al., 2019; Tan et al., 2020; Lee et al., 2021).

Cmpd	Structure	Ki (nM)					
		D_1_R	D_2_R	D_3_R	D_4_R	D_5_R	5-HT_2C_
(1*S*,2*S*)-**17a**	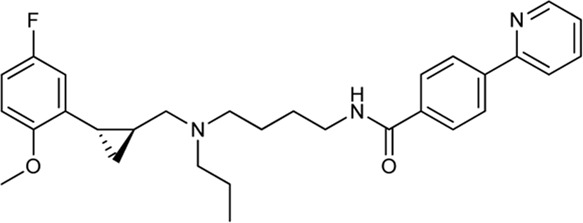	1,071	1,230	3.8	851	>5,000	50.1
(1*R*,2*R*)-**17b**	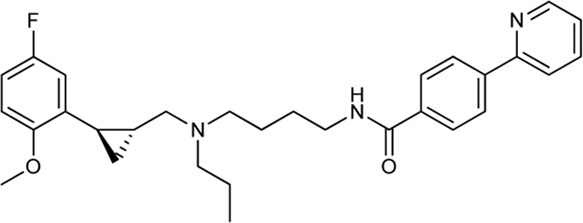	4,898	1,349	4.1	575	>5,000	1,122
(1*S*,2*S*)-**18a**	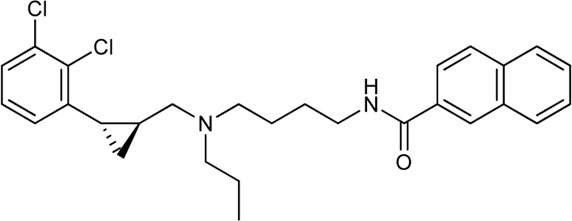	1,047	1,148	20.8	776	>5,000	138
(1*R*,2*R*)-**18b**	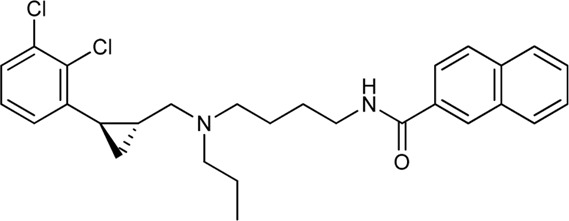	1,288	676	4.4	813	>5,000	513
(1*S*,2*S*)-**19a**	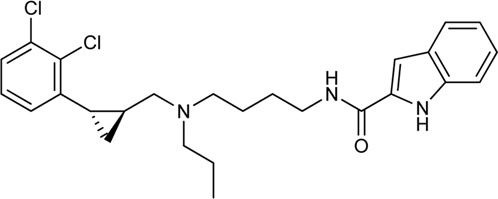	1,122	992	12.8	676	>5,000	61.7
(1*R*,2*R*)-**19b**	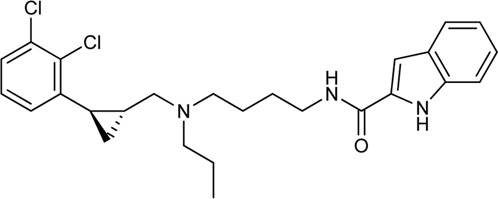	1,380	537	2.2	1,047	>5,000	513
(1*S*,2*S*)-**20a**	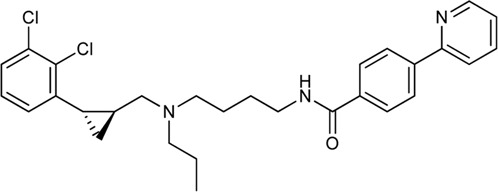	2344	1,023	5.3	912	>5,000	44.7
(1*R*,2*R*)-**20b**	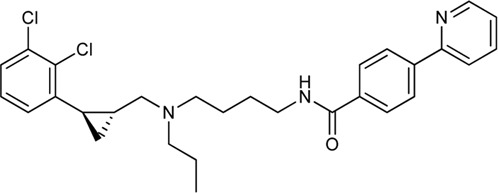	1,349	550	1.5	676	>5,000	417
**Cmpd**	**Structure**	**D**_**2**_ **R K**_**i**_ **(nM)**	**D**_**3**_ **R K**_**i**_ **(nM)**	**D**_**4**_ **R K**_**i**_ **(nM)**	**D**_**2**_ **R/D**_**3**_ **R**	**D**_**4**_ **R/D**_**3**_ **R**	
**24**	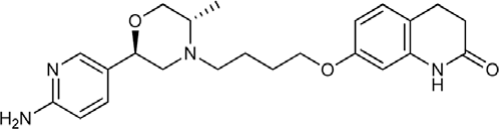	2600	24200	ND	0.110	ND	
**25**	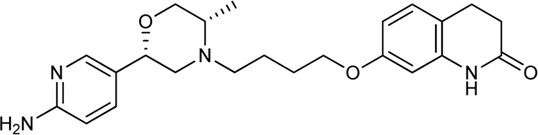	34.6	31.2	ND	1.1	ND	
**27**	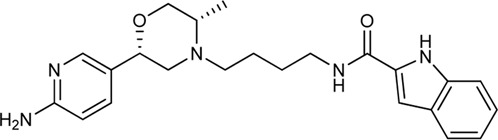	134	5.96	357	22.5	59.9	
**28a**	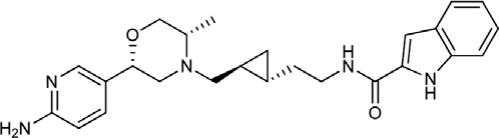	87.8	1.85	286	47.5	155	
**28b**	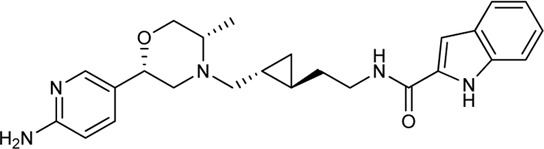	831	282	2930	2.95	10.4	
**39**	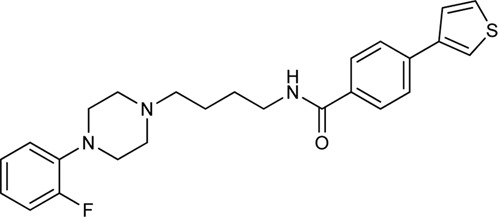	648	1.4	-	467	-	

Battiti and co-workers performed a SAR analysis combining two PPs for the synthesis of bitopic compounds; one is a selective dopamine agonist PF-592379 (Allerton et al., 2005; Ackley, 2008) and the other is PD-128907, which is a D_2_R/D_3_R agonist. **(** Supplementary Table S4) They concluded that the structural features of PD-128907 avoided the construction of bitopic compound. Therefore, they focused to PF-592379 to synthesize D_2_R/D_3_R active bitopic compounds. Here we discuss a representative example for different SPs (Supplementary Table S4). D_2_R and D_3_R binding data clearly show that the (S,S) enantiomer of the PP is more favourable for receptor binding. The (S,S) enantiomer already plays a prominent role in PP (**22**, **23**), with a 3-fold activity difference between the enantiomers. The same effect can be observed when using tetrahydroisoquinoline (**24**,**25**) or indole (**26**,**27**) SP, although here the difference in activity at the D_3_ receptor is about 100-fold (Table 1, Supplementary Table S4). Compound **27** show a 22.5-fold subtype selectivity towards D_3_R that is due to the SP moiety. Next the authors investigated the effect of the linker. Changing the original cyclopropylethyl linker (Supplementary Table S4) for the racemic derivative (**rac-trans-28**) resulted in 37.3-fold selectivity towards D_3_R. Separating the enantiomers, (1S, 2R)-trans-cylopropyl stereochemistry (**28a**) showed D_3_R K_i_ of 1.85 nM and an unprecedented 47.5-fold selectivity for D_3_R over D_2_R (D_2_R Ki = 87.8 nM), while the other enantiomer (**28b**) has much weaker activity coupled with poor selectivity (Battiti et al., 2019). Finally, two additional linkers were used (**31**, **32**) that are widely used among D_3_R bitopic compounds including several high selectivity partial agonists or antagonists (Kumar et al., 2016; Michino et al., 2017; Verma et al., 2018). Compound **31** showed reduced affinity compared to **28a**, inferring that the hydroxyl group on the linker is optimal for antagonism but cannot be directly transferred to the agonist binding mode due to different receptor conformations in the active and inactive states. Compound **32** shows good affinity but neither affinity nor selectivity reaches that of **28a**. Compounds **31**, **32**, **28a** were tested at MOR. For **32** there is a decrease in affinity at the dopamine receptor but the weak subtype selectivity is retained, however there is a 22.9-fold increase in activity at the MOR receptor (Supplementary Table S4) (Battiti et al., 2020). The same group synthesized a number of eticlopride analogues using different SPs in the 2-N or 4-C position of pyrrolidine *via* lycerol (Battiti et al., 2020). They found that O-alkylated analogues had better affinity for D_2_ and D_3_ receptors than the N-substituted derivatives. In BRET assays, these compounds exhibited antagonist or very weak partial agonist behaviour. Docking studies revealed that the SPs of the O-alkylated analogues form aromatic stacking interactions with conserved residues His6.55 and Tyr7.35 both in the D_2_ and D_3_ receptors, while the SPs of the N-alkylated derivatives extend towards the extracellular site that is less conserved (Shaik et al., 2021).

N-phenylpiperazine analogues were used extensively for constructing bitopic ligands against dopamine receptors. Lee et al. synthesized and evaluated a series of N-phenylpiperazine analogues substituted with 3-thiophen and 4-thiazolylphenylfluoride (Supplementary Table S5). They identified several ligands that bind with high affinity to D_3_R and exhibit considerable selectivity towards D_2_R. Comparison of the binding results of compounds **33–38** and **39–44** suggests that **39–44** binds to D_3_R but not to D_2_R. The replacement of the thiophene ring by a thiazole ring (**45–50**) led to a decrease in receptor binding selectivity. Compound **39** (Table 1) possessed the highest D_3_R affinity (K_i_ = 1.4 nM) and 450-fold selectivity that nominated this compound for *in vivo* testing. Intraperitoneal administration of **39** led to a significant reduction in DOI-dependent head twitch response in mice and a reduction in AIM scores in dyskinetic hemiparkinsonian rats. These data suggest that compound **39** is able to cross the blood-brain barrier and achieves therapeutic concentrations (Lee et al., 2021).

Starting from the 5-HT_2A_ receptor-bound structure of aripiprazole and cariprazine Chen et al. designed D_2_/D_3_ receptor ligands with no significant 5-HT_2A_ affinity (Chen et al., 2021). The authors suggested that the unusal “upside-down” binding mode (Figure 3A) might affect the observed selectivity. According to the structural rearrangements, the location of the SP of aripiprazole in the exosite is important for its signal transduction efficiency. In the interest of identifying residues critical for efficacy, the exosite sequence of the 5-HT_2A_ and D_2_ receptors was aligned, with an important difference between the two found at position 5.51, which is Leu in 5-HT_2A_R and Phe in D_2_R. Mutations in D_2_R demonstrated that substitution of F202^5.51^ with Leu or Ala reduces the G-protein activity and β-arrestin2 recruitment of aripiprazole. In addition, a derivative of aripiprazole substituted with benzothiazole for the dihydroquinoline ring of D_2_R had reduced efficiencies of both G protein activity and β-arrestin2 recruitment. Substitution of L247^5.51^F in 5-HT_2A_R did not increase the efficacy of aripiprazole. The results suggest that aripiprazole may stabilize different conformations of TM5 and TM6 between the two receptors. Alignment of 5-HT_2A_R and D_2_R structures (active and inactive) shows that activation of 5-HT_2A_R requires a larger downstream swing of W6.48 from the CWxP motif than that observed for D_2_R activation. In the 5-HT_2A_R, dihydroquinoline is located deeper in the binding pocket interacting with W336^6.48^, restricting its movement, whereas it can move gently upon D_2_R receptor activation. Similar observations were made for cariprazine. Here, the dynamic coupling between F/L5.51, W6.48 and the PIF motif by the exosite may partly explain why the compounds tested have different efficacies at 5-HT_2A_R and D_2_R receptors. Compared with inactive and active D_2_R constructs, the 5-HT_2A_R-aripiprazole complex in the extracellular compartment shows inward movement of TM6, TM7, and ECL2 toward the seven transmembrane cores. These rearrangements suggest that the 5HT_2A_R affinity of the bitopic compounds can be reduced by increasing the size of the PP. Changing the arylpiperazine PP to aza-ergoline the authors identified IHCH7009 (D_2_R K_i_ = 33.65 nM, 5-HT_2A_R K_i_ = 3639.15 nM), IHCH7010 (D_2_R K_i_ = 9.03 nM, 5-HT_2A_R K_i_ = 906.78 nM) and IHCH7041 (D_2_R K_i_ = 50.64 nM, 5-HT_2A_R K_i_ = 2371.37 nM) all with very weak 5-HT_2A_R affinity. IHCH7041 retains partial agonism of D_2_R while IHCH7009 and IHCH7010 are full D_2_R agonists (Chen et al., 2021).

Kling et al. investigated the neurotensin receptor type (NTS) 1 receptor crystal structures (White et al., 2012; Egloff et al., 2014) and found that an allosteric binding site was saturated at the C-terminus of NT (8–13). Following sequence analysis, they confirmed that there is a difference between NTS_1_R (Arg149^3.32^) and NTS_2_R (His115^3.32^) that may allow for subtype selectivity. Several bitopic ligands of type NT (8–13) were synthesized (Table 2) and compounds (**51–56**) showed a promising trend in the NTS_1_R selectivity. The best compound (**54**) has K_i_ value of 1.3 nM associated with 26-fold selectivity towards NTS_2_R. Homology modelling and MD simulations confirmed that the compounds bind in a bitopic mode, with NT (8–13) occupying the orthosteric binding site and the amino acid extension occupying the secondary binding site. These results provide a promising starting point for the design of NTS_1_R selective agonists (Kling et al., 2019).

**TABLE 2:** NTS1 and NTS2 receptor-binding data for bitopic ligands (Kling et al., 2019).
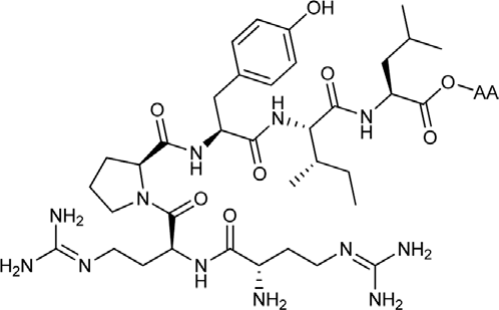

Cmpd	NT (8–13)-AA	K_i_ (nM)		NTS2/NTS1	IP acc. Assay	
		**NTS_1_** **nM±SEM**	**NTS**_**2**_ **nM±SEM**		**EC**_**50**_ **nM±SEM**	**Efficacy** **%** **±** **SEM**
**NT(8–13)**		0.24 ± 0.048	1.2 ± 0.25^[h]^	5.0	0.74 ± 0.20	100%
**51**	NT (8–13)-Gly-OH	6.8 ± 4.5	53 ± 21	7.8	18 ± 4	98 ± 2%
**52**	NT (8–13)-Ser-OH	3.3 ± 1.7	58 ± 28	18	37 ± 16	98 ± 5%
**53**	NT (8–13)-Phe-OH	0.91 ± 0.49	12 ± 4.0	13	150 ± 22	100 ± 5%
**54**	NT (8–13)-Tyr-OH	1.3 ± 0.38	34 ± 9.4	26	110 ± 26	95 ± 10%
**55**	NT (8–13)-hTyr-OH	1.5 ± 0.65	37 ± 9.1	25	24 ± 5	92 ± 8%
**56**	NT (8–13)-*meta*-Tyr-OH	2.1 ± 0.4	44 ± 23	21	34 ± 7	94 ± 4%

### Functional Selectivity

Advances in GPCR structural biology and pharmacology have opened up new opportunities for functional drug design. Modulation of GPCRs through allosteric binding sites can alter receptor structure, dynamics and function, resulting in increased spatial and temporal variation. One important aspect of these changes is functional selectivity or otherwise termed biased signalling. Biased signalling can contribute to the enhancement of the intended effect, but can also cause side effects, so one of the most intriguing areas of current research is investigating the functional character of the ligands in different signalling pathways (Hauser et al., 2017).

**FIGURE 6:**
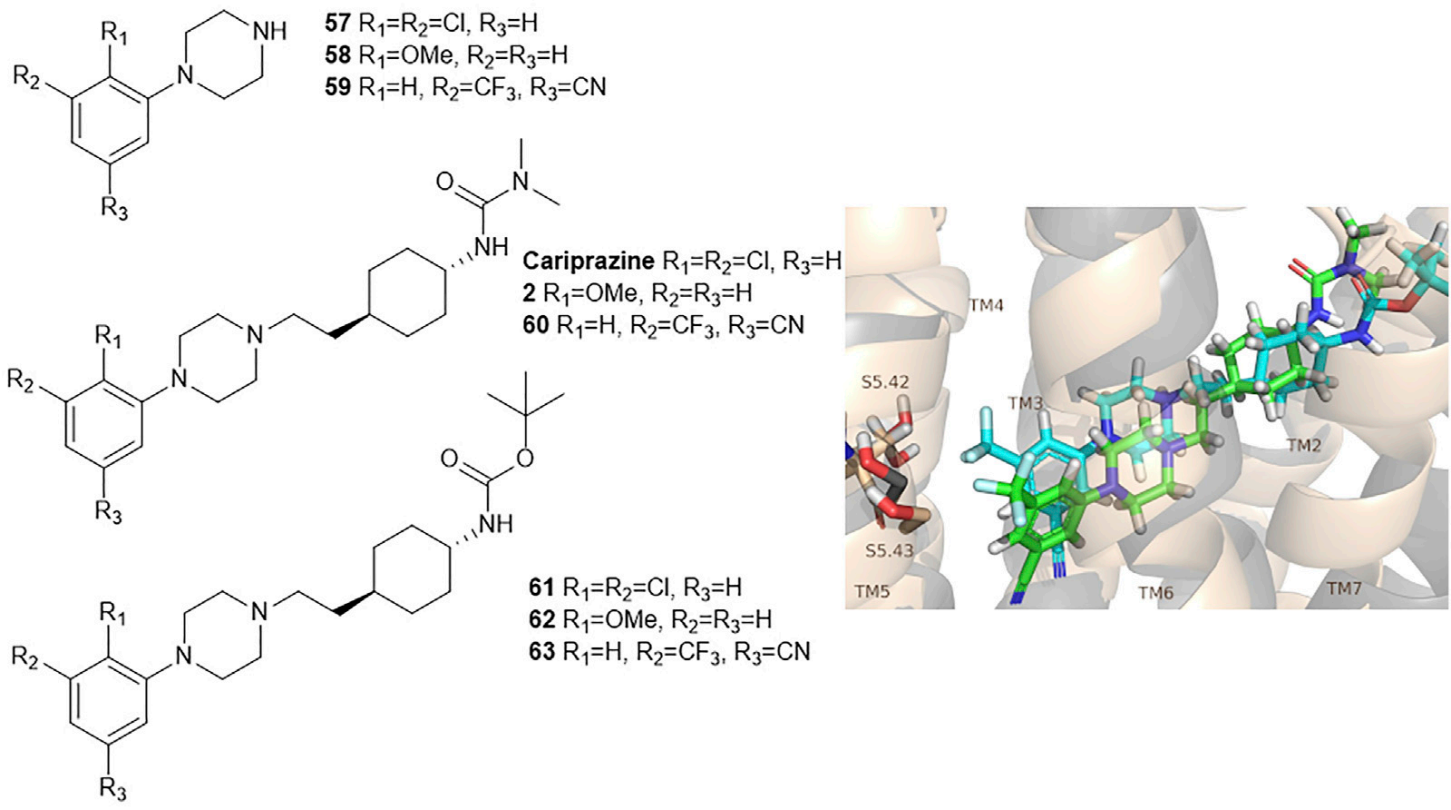
D_2_R and D_3_R ligands with designed functional profile (Egyed et al., 2020). The binding mode of compound **60** and **63** was extracted from the MD simulations. The simulations revealed that the SP motif influence the position of the PP and that might be linked to the observed different functional profile. The figure representing the binding mode is reproduced from the TOC Figure of our original article Egyed, A et al. Controlling Receptor Function from the Extracellular Vestibule of G-Protein Coupled Receptors. Chem. Commun. 2020, 56 (91), 14167–14170.

Egyed at al. reported a systematic study exploring the extracellular SBP to fine-tune the functional profile of D_2_R and D_3_R ligands. Introduction of the SP increased affinity at both D_2_ and D_3_ receptors for each ligand. The study demonstrated that the G_i/o_ and β-arrestin pathways can be specifically modulated from the extracellular vestibule incorporating different SPs to the ligands. Molecular dynamics simulations revealed that G-protein signalling could be linked to the orientation of the PP that is influenced by the SBP binding part of the bitopic compounds (Figure 6). Three PPs and two SPs (Figure 6) were tested using an ethylcyclohexyl linker in analogy to cariprazine. In the G_i/o_-mediated signalling pathway, dichlorophenylpiperazine (**57**) (PP 1) was a partial agonist on both D_2_R and D_3_R (Table 3). Application of N,N-dimethylurea (SP 1) (**cariprazine**) also resulted in a partial agonist with significantly increased potency (D_2_R pEC_50_ = 8.85 nM, E_max_ = 77.4%, D_3_R pEC_50_ = 8.58 nM E_max_ = 27%). The use of the OtBu motif (SP 2) (**61**) led to a full agonist, the potency on D_2_R was superior to that on D_3_R. For 2-methoxyphenylpiperazine (**2, 58, 62**) (PP 2), no prominent change was observed, all were partial agonists. The 3-(piperazin-1-yl)-5-(trifluoromethyl)benzonitrile (**59**) (PP 3) with the N,N-dimethylurea SP (**60**), showed antagonist effects on the G protein coupled signalling pathway of D_2_R and D_3_R, with an increase in potency. Interestingly, incorporating SP 2 (**63**) turned the function of PP to a weak partial agonist at both receptors. These results suggest that PP and SP affect functionality together. In the β-arrestin signalling pathway, compounds with SP 2 achieve the largest increase in E_max_ values, while this was lower for cariprazine. (Table 3). This suggests that cariprazine shows a significant bias towards the G-protein controlled pathway on D_2_R. In all cases, the bitopic compounds with 2-methoxyphenylpiperazine PP (**2, 58, 62**) exhibited antagonist behaviour in contrast to the partial agonism observed in the G-protein coupled signalling pathway. The antagonistic behaviour of **59** was also preserved in the β-arrestin signalling pathway; following the previous trends introduction of any SP led to an increase in pIC_50_ values here as well. In general, the efficacy data measured at both receptors followed similar trends in both modalities as the receptor affinities (Egyed et al., 2020).

**TABLE 3:** Functional activities (pIC_50_ or pEC_50_ and maximal efficacy (Emax) values with s.d. values in parentheses) measured for the G-protein mediated and β-arrestin mediated pathway of the hD_2_ and hD_3_ receptor (Egyed et al., 2020).

hD_2_R	G-protein mediated pathway			β-Arrestin mediated pathway		
	H	SP 1	SP 2	H	SP 1	SP 2
**PP 1**	**57** EC_50_ < 4.3 uM E_max_ = 45.6% (3) partial agonist	**Cariprazine** pEC_50_ = 8.85 (0.1) Gao et al. (2014) E_max_ = 77.4% (7) partial agonist	**61** pEC_50_ = 8.64 (0.22) E_max_ = 99.4% (2) full agonist	pEC_50_ = 3.85 (0.12) E_max_ = 7% (1) partial agonist	pEC_50_ = 9.69 Gao et al. (2014)E_max_ = 13.9% partial agonist	pEC_50_ = 8.40 (0.17) E_max_ = 26% (2) partial agonist
**PP 2**	**58** pIC_50_ = 6.4 (1.0) Newman et al. (2012) E_max_ = 14% (1) partial agonist	**2** pEC_50_ = 8.62 (0.07) E_max_ = 82.7% (3) partial agonist	**62** pIC_50_ = 8.42 (0.18) E_max_ = 78.7% (4) partial agonist	pIC_50_ = 5.03 (0.12) antagonist	pIC_50_ = 8.08 (0.05) antagonist	pIC_50_ = 7.63 (0.10) antagonist
**PP 3**	**59** pIC_50_ = 4.72 (0.78) antagonist	**60** pIC_50_ = 6.10 (0.13) antagonist	**63** EC_50_ > 50 uM E_max_ = 25.4% (4) partial agonist	pIC_50_ = 5.89 (0.13) antagonist	pIC_50_ = 7.71 (0.10) antagonist	pIC_50_ = 7.23 (0.12) antagonist

**hD**_**3**_ **R**	G-protein mediated pathway			β-arrestin mediated pathway		
	**H**	**SP 1**	**SP 2**	**H**	**SP 1**	**SP 2**
**PP 1**	pEC_50_ = 7.50 (0.34) E_max_ = 72% (12) partial agonist	pEC_50_ = 8.58 Kiss et al. (2010) E_max_ = 27% Kiss et al. (2010) partial agonist	pEC_50_ = 8.09 (0.13) E_max_ = 94% (7) full agonist	30% (5) in 80 μM partial agonist	pEC_50_ = 8.32 Frank et al. (2018) E_max_ = 32% Frank et al. (2018)partial agonist	pEC_50_ = 8.42 (0.21) E_max_ = 61% (6) partial agonist
**PP 2**	pEC_50_ = 6.12 (0.17) E_max_ = 11% (4) partial agonist	pEC_50_ = 8.43 (0.51) E_max_ = 11% (3) partial agonist	pEC_50_ = 8.63 (0.13) E_max_ = 15% (6) partial agonist	pIC_50_ = 4.83 (0.30) antagonist	pIC_50_ = 7.92 (0.10) antagonist	pIC_50_ = 7.52 (0.20) antagonist
**PP 3**	pIC_50_ = 5.01 (0.17) antagonist	pIC_50_ = 7.56 (0.23) antagonist	pEC_50_ = 7.53 (0.34) E_max_ = 15% (3) partial agonist	pIC_50_ = 5.44 (0.15) antagonist	pIC_50_ = 8.04 (0.32) antagonist	pIC_50_ = 7.86 (0.21) antagonist

The bold values indicate the number of compounds.

High affinity binders, such as **39, 40, 41, 42, 49** (Supplementary Table S5) were also tested for their efficacy on D_3_R, both by examining forskolin-dependent inhibition of adenylyl cyclase and by measuring β-arrestin binding. Compounds **42** and **49** were found antagonists in both assays. Compound **41** display functional selectivity, being a weak partial agonist in the adenylyl cyclase assay and a very weak partial agonist/antagonist in the β-arrestin binding assay. Compounds **39** and **40** exhibit weak partial agonism in both the adenylyl cyclase inhibition and β-arrestin binding assays (Lee et al., 2021).

Investigating pure enantiomeric forms of compounds **17–20** (Supplementary Table S3) Tan et al. showed that the (R,R) enantiomers (**17b-20b**) have a better affinity for D_3_R than (S,S) (**17a-20a**), with the exception of compound **17**, which had an identical affinity for both of the enantiomers (**17a, 17b**) (Tan et al., 2020). The (R,R) isomers (**17b-20b**) showed weaker affinity (3–20-fold) towards 5-HT_2C_R than their (S,S) counterparts (**17a-20a**). The data suggest that D3R is less sensitive to conformational changes than the 5-HT_2C_ receptor. Functional studies were also performed with the **17a,b-20a,b** (Table 4). Compounds **18–20** were all full or partial agonist on D_3_ receptors, whereas for 5-HT_2C_R the (S,S) enantiomers (**18a-20a**) are weak partial agonists, whereas the (R,R) enantiomers (**18b-20b**) are weak antagonists. Compared to the binding assay, functional results indicate greater selectivity towards D_3_R. Furthermore, these compounds showed only very weak partial agonism at 5-HT_2A_R and no affinity at 5-HT_2B_R. The two enantiomers of compound **17** exhibit opposite behaviour, while **(1R,2R)-17b** was a potent agonist (EC_50_ = 3.6 nM, E_max_ = 77.9%), **(1S,2S)-17a** was an antagonist on D_3_R with a K_i_ of 16.7 nM, and both derivatives were weak antagonists with micromolar activity on 5-HT_2C_ receptor. Docking studies suggested a difference between the two compounds (**17a,17b**) in the orientation of PP. In the case of the agonist **(1R,2R)-17b**, the 2-methoxy group is deep in the OBP and forms hydrophobic interactions with residues C114^3.36^, S196^5.46^, and F346^6.52^. In the case of the antagonist **(1S,2S)-17a**, the 2-methoxy group flips out to the extracellular side and the cyclopropane linker between the benzene ring and the protonated N overlays perfectly with the amide linker of eticlopride, which is not present in the agonist. Compounds **(1S,2S)-17a**, **(1R,2R)-18b**, **(1R,2R)- 19b**, and **(1R,2R)-20b** were inactive in the Tango assay on D_3_R, indicating their preference for the G-protein signalling pathway. For further profiling **(1R,2R)-17b** and **(1R,2R)-19b** were tested on 29 other aminergic GPCRs that confirmed their good selectivity for D_3_R (Tan et al., 2020).

**TABLE 4:** Functional Data of compounds at D_3_R and 5-HT_2C_ (All compounds were tested as HCl salts. For agonist activity, Emax values are shown in brackets. NT, not tested.).

Cmpd	D_3_R Gi	D_3_R Tango	5-HT_2C_G_q_ (Ca2+)
**(1*R*,2*R*)-17b**	EC_50_ = 3.58 nM (77.9%b)	EC_50_ = 126.4 nM (50.2%)	antagonist IC_50_ = 14.5 μM
**(1*S*,2*S*)-17a**	no agonism; antagonist: *K*i = 16.7 nM	NT	antagonist IC_50_ = 0.86 μM
**(1*R*,2*R*)-18b**	EC_50_ = 177.5 nM (71.7%)	9.2% at 3 μM	antagonist IC_50_ = 16.1 μM
**(1*S*,2*S*)-18a**	EC_50_ = 99.2 nM (83.4%)	44.4% at 3 μM	agonist EC_50_ = 3538 nM (30.3%)
**(1*R*,2*R*)-19b**	EC_50_ = 87.0 nM (40.7%)	<5% at 3 μM	antagonist: IC_50_ > 30 μM
**(1*S*,2*S*)-19a**	EC_50_ = 142.8 nM (63.4%)	EC_50_ = 1,000.2 nM (27.1%)	agonist EC_50_ = 2549 nM (44.2%)
**(1*R*,2*R*)-20b**	EC_50_ = 12.5 nM (68.1%)	3.1% at 3 μM	antagonist IC_50_ = 10.1 μM
**(1*S*,2*S*)-20a**	EC_50_ = 29.6 nM (96.2%)	EC_50_ = 11086 nM (119.1%)	agonist EC_50_ = 738.3 nM (51.9%)

Yan et al. also used PCPMA analogues as PP, with propyl, butyl, pentyl, or cyclohexylethyl linkers, and SP groups taken from aripiprazole, brexipirazole, and cariprazine, respectively. The synthesized library was measured in D_2_R binding, D_2_R G_i_ and D_2_R β-arrestin BRET assays (Table 5). The starting compound (**64)** exhibits good affinity (K_i_ = 61.9 nM) and partial agonist activity in both G_i_ (EC_50_ = 49.0 nM, E_max_ = 25%) and β-arrestin (EC_50_ = 67.6 nM, E_max_ = 30%) BRET assays. In comparison, replacement of SP with quinolone (**65)** increased the potency two-fold with unchanged binding. Changing the linker to propyl (**66**,**67**) led to a small decrease in binding affinity but an increase in efficacy (∼10 nM EC_50_ values and E_max_ values higher than 50%). Lengthening the linker to 5C units (**68,69**) led to a decrease in binding affinity and functional activity. The cariprazine-like SP (dimethylamine) and linker (cyclohexyl) with this PP did not show significant activity. The best compound from this series (**70**) has very potent partial agonist character in both G_i_ BRET (EC_50_ = 8.45 nM, E_max_ = 68%) and β-arrestin2 recruitment assays (EC_50_ = 9.49 nM, E_max_ = 16%), with a much lower E_max_ in the latter. The significant difference between binding affinity and potency for many of these compounds likely reflects the use of an antagonist radioligand [(^3^H)-N-methylspiperone] in the competitive binding assay, from which an agonist ligand tends to show much lower apparent binding affinity. Attempts have been made to use several PPs but these have been shown to give significantly worse results than the methoxy derivative. In the case of isoquinoline and tetrahydroisoquinoline SP, it was not practical to use the dichlorophenyl motif in the PP (**71,72**). The best results were obtained with derivatives containing halogen in the meta position on the phenyl group of PP and methoxy in the ortho position **(73a,b-76a,b**). Pure forms of the enantiomers were also investigated. The majority of the fluorinated derivatives (**(1S,2S)-42a, (1R,2R)-73b, (1S,2S)-74a**) showed K_i_ values below 50 nM on binding assay and EC_50_ values below 20 nM in both G_i_ and β-arrestin2 BRET assays. The same trend was observed for the chlorinated derivatives [**(1S,2S)-75a**,**(1S,2S)-76a**]. Higher E_max_ was observed for the halogenated derivatives in the G_i_ signal transduction than in the β-arrestin. After separation of the enantiomers, it was confirmed that the (S,S)-isomers were more efficient in D_2_R binding and functional assay. The (R,R) compounds exhibit partial agonist behaviour and the E_max_ values are higher for G_i_ signaling. The selectivity of the compounds [**(1S,2S)-73a, (1S,2S)-74a, (1S,2S)-75a, (1S,2S)-76a**] was investigated on D_1_R, D_2_R, D_4_R, D_5_R, 5-HT_1A_R, 5-HT_2A_R, and 5-HT_2C_R, with low selectivity observed towards the D_3_ receptor and potent activity on the 5-HT_1A_ receptor, and good or acceptable selectivity on the other receptors (Table 6). In the case of D_3_R, these compounds showed weak partial agonist activity in both G_o_ and β-arrestin2 BRET assays, albeit with different efficacies. For the 5-HT_1A_ receptor, all four compounds (**(1S,2S)-73a, (1S,2S)-74a, (1S,2S)-75a, (1S,2S)-76a**) were similar partial agonists in G_i_ BRET assays. The lack of selectivity over D_3_R and 5-HT_1A_R should not be a concern for these compounds, as both D_3_R and 5-HT_1A_R have been shown to be involved in the therapeutic effects of some antipsychotics. Overall, these four compounds have shown an interesting pharmacological profile (Yan et al., 2021).

**TABLE 5:** Pharmacological profiling of compounds (D2R binding and functional activity) (Yan et al., 2021).

Cmpd	Structure	D_2_R binding K_i_ nM (pK_i_±SEM)	D_2_R G_αi1_ BRET EC_50_ nM (E_max_%) (pEC_50_ ± SEM)	D_2_R β-arrestin2 BRET EC_50_ nM (E_max_%) (pEC_50_ ± SEM)
**64**	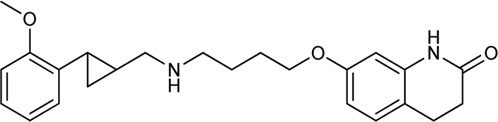	61.9 (7.21 ± 0.04)	49.0 (25 ± 2%) (7.31 ± 0.09)	67.6 (30 ± 1%) (7.17 ± 0.07)
**65**	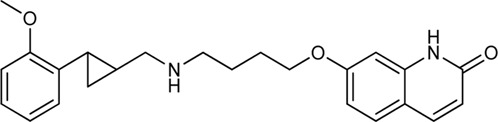	59.9 (7.22 ± 0.13)	26.3 (52 ± 1%) (7.58 ± 0.08)	32.4 (53 ± 2%) (7.49 ± 0.14)
**66**	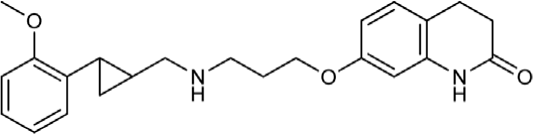	125.7 (6.90 ± 0.08)	9.30 (58 ± 3%) (8.03 ± 0.01)	10.0 (52 ± 1) (8.00 ± 0.11)
**67**	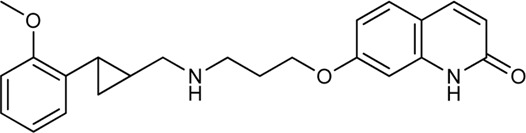	155.7 (6.81 ± 0.03)	11.2 (65 ± 3%) (7.95 ± 0.04)	7.08 (60 ± 1%) (8.15 ± 0.12)
**68**	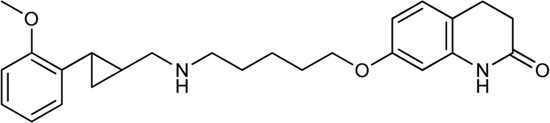	259.2 (6.59 ± 0.05)	891.2 (12 ± 1%) (6.05 ± 0.42)	416.9 (14 ± 4%) (6.38 ± 0.64)
**69**	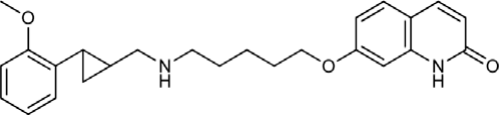	217.8 (6.66 ± 0.08)	77.6 (18 ± 1%) (7.11 ± 0.12)	190.6 (19 ± 1%) (6.72 ± 0.49)
**70**	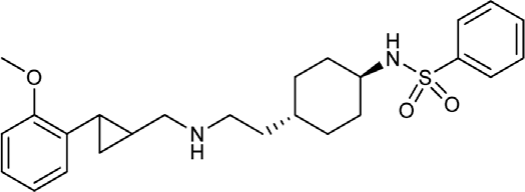	977.2 (6.01 ± 0.11)	8.45 (68 ± 1%) (8.07 ± 0.11)	9.49 (16 ± 1%) (8.02 ± 0.06)
**71**	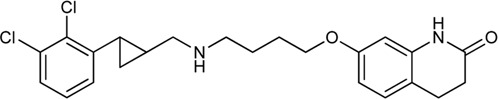	244.3 (6.61 ± 0.07)	34.8 (51 ± 5%) (7.46 ± 0.10)	94.0 (39 ± 4%) (7.03 ± 0.20)
**72**	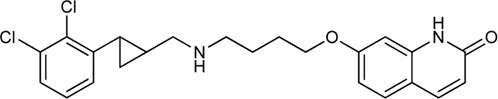	128.1 (6.89 ± 0.112)	14.73 (66 ± 3%) (7.83 ± 0.12)	27.6 (33 ± 1%) (7.56 ± 0.09)
**(1S,2S)-73a**	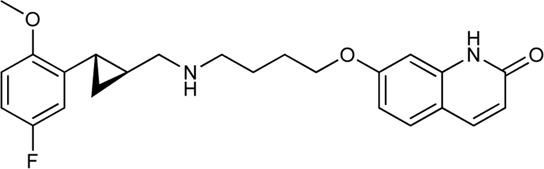	20.8 (7.68 ± 0.06)	9.43 (29 ± 3%) (8.03 ± 0.05)	3.63 (18 ± 1%) (8.44 ± 0.17)
**(1R,2R)-73b**	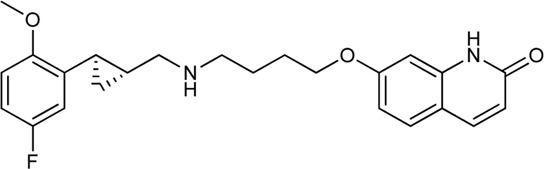	43.8 (7.36 ± 0.07)	12.9 (13 ± 3%) (7.89 ± 0.14)	1.86 (10 ± 2%) (8.71 ± 0.15)
**(1S,2S)-74a**	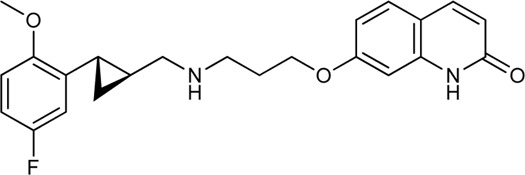	6.58 (8.18 ± 0.04)	4.12 (55 ± 2%) (8.39 ± 0.08)	4.66 (29 ± 1%) (8.33 ± 0.15)
**(1R,2R)-74b**	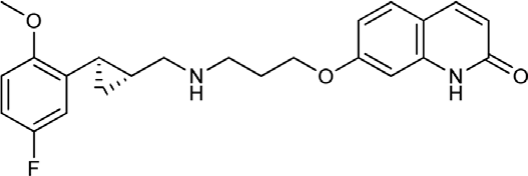	362.5 (6.44 ± 0.07)	62.0 (7 ± 1%) (7.21 ± 0.16)	14.7 (17 ± 1%) (7.83 ± 0.12)
**(1S,2S)-75a**	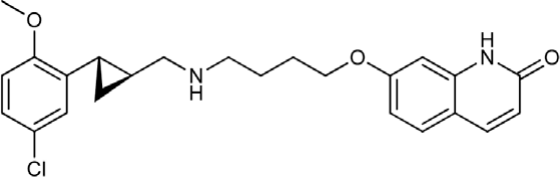	11.5 (7.94 ± 0.07)	8.9 (40 ± 2%) (8.05 ± 0.04)	2.50 (20 ± 1%) (8.60 ± 0.10)
**(1R,2R)-75b**	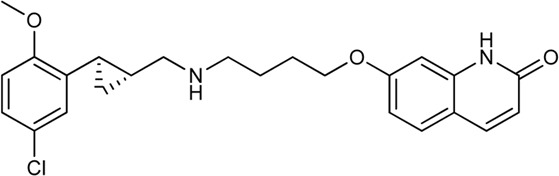	30.1 (7.52 ± 0.02)	NT	NT
**(1S,2S)-76a**	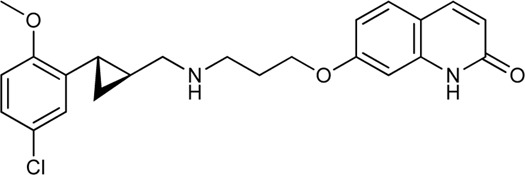	12.8 (7.89 ± 0.05)	3.41 (71 ± 3%) (8.47 ± 0.08)	8.30 (47 ± 2%) (8.08 ± 0.06)
**(1R,2R)-76b**	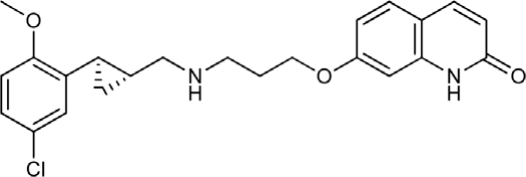	317.0 (6.50 ± 0.04)	197.2 (41 ± 5%) (6.71 ± 0.05)	70.1 (18 ± 3%) (7.15 ± 0.15)

**TABLE 6:** Binding and functional datas for enantiomer selective lingands (Yan et al., 2021).

*K*_i_, nM (p*K*_i_±SEM)							
Cmpd	D_1_R	D_2_R	D_3_R	D_4_R	5-HT_1A_	5-HT_2A_	5-HT_2C_
**(1S,2S)-73a**	>10,000	20.8 (7.68 ± 0.06)	73.6 (7.13 ± 0.26)	122.3 (6.91 ± 0.23)	34.5 (7.46 ± 0.30)	1,411 (5.85 ± 0.18)	122.3 (6.91 ± 0.23)
**(1S,2S)-74a**	>10,000	6.58 (8.18 ± 0.04)	22.6 (7.65 ± 0.33)	304.6 (6.52 ± 0.34)	19.0 (7.72 ± 0.16)	519.6 (6.28 ± 0.04)	304.6 (6.52 ± 0.34)
**(1S,2S)-75a**	>10,000	11.5 (7.94 ± 0.07)	37.6 (7.43 ± 0.29)	373.0 (6.43 ± 0.21)	30.3 (7.52 ± 0.05)	2093 (5.68 ± 0.10)	373.0 (6.43 ± 0.21)
**(1S,2S)-76a**	>10,000	12.8 (7.89 ± 0.05)	33.9 (7.47 ± 0.28)	604.0 (6.22 ± 0.09)	32.8 (7.48 ± 0.13)	1,160 (5.94 ± 0.12)	604.0 (6.22 ± 0.09)
**Aripiprazole**	1,146 (5.94 ± 0.06)	2.13 (8.67 ± 0.03)	4.02 (8.40 ± 0.10)	100.8 (7.00 ± 0.19)	13.3 (7.88 ± 0.01)	39.6 (7.40 ± 0.03)	95.4 (7.02 ± 0.08)
**cariprazine**	3414 (5.47 ± 0.11)	1.45 (8.84 ± 0.07)	0.27 (9.57 ± 0.21)	507.0 (6.30 ± 0.16)	4.01 (8.40 ± 0.06)	219.4 (6.66 ± 0.05)	198.2 (6.70 ± 0.04)
**haloperidol**	NT	6.33 (8.20 ± 0.08)	22.7 (7.64 ± 0.18)	26.3 (7.58 ± 0.07)	NT	NT	NT
**LE300**	2.93 (8.53 ± 0.13)	NT	NT	NT	NT	NT	NT
**5-HT**	NT	NT	NT	NT	6.50 (8.19 ± 0.19)	79.1 (7.10 ± 0.07)	26.4 (7.58 ± 0.07)

**Cmpd**	**D**_**2**_ **R Gα**_**oa**_ **BRET EC**_**50**_ **, nM** (***E***_**max**_ **%) (pEC**_**50**_ **± SEM)**	**D**_**3**_ **R Gα**_**oa**_ **BRET EC**_**50**_ **, nM** (***E***_**max**_ **%) (pEC**_**50**_ **± SEM)**	**D**_**3**_ **R β-arrestin2 BRET EC**_**50**_ **, nM** (***E***_**max**_ **%) (pEC**_**50**_ **± SEM)**	**5-HT**_**1A**_ **G**_**αi1**_ **BRET EC**_**50**_ **, nM** (***E***_**max**_ **%) (pEC**_**50**_ **± SEM)**
**(1S,2S)-73a**	7.18 (44 ± 2%) (8.14 ± 0.25)	5.14 (19 ± 4%) (8.29 ± 0.34)	52.91 (17 ± 5%) (7.28 ± 0.21)	95.94 (58 ± 2%) (7.02 ± 0.13)
**(1S,2S)-74a**	1.60 (66 ± 3%) (8.80 ± 0.13)	117.6 (23 ± 7%) (6.93 ± 0.21)	9.84 (18 ± 3%) (8.01 ± 0.07)	51.96 (49 ± 2%) (7.28 ± 0.10)
**(1S,2S)-75a**	6.45 (57 ± 2%) (8.19 ± 0.11)	97.65 (31 ± 2%) (7.01 ± 0.20)	37.35 (23 ± 4%) (7.43 ± 0.11)	45.43 (38 ± 2%) (7.34 ± 0.27)
**(1S,2S)-76a**	2.03 (77 ± 2%) (8.69 ± 0.07)	129.3 (54 ± 5%) (6.89 ± 0.05)	12.89 (46 ± 1%) (7.89 ± 0.13)	58.75 (45 ± 3%) (7.23 ± 0.11)
**Quinpirole**	1.18 (97 ± 2%) (8.93 ± 0.02)	1.97 (100 ± 2%) (8.71 ± 0.02)	3.31 (101 ± 1%) (8.48 ± 0.08)	NT
**5-HT**	NT	NT	NT	6.29 (98 ± 2%) (8.20 ± 0.09)

Schramm et al. investigated the effect of bitopic compounds on muscarinic acetylcholine receptors. Carbachol (CCh) PP was cross-linked to allosteric ligands by linkers of different lengths (1C, 3C, 5C, 8C). The benzoimidazole-piperidine moiety of TBPB [1-(1′-(2-tolyl)-1,4′-bipiperidin-4-yl)-1H-benzo(d)imidazol-2(3H)-one], a known selective bitopic M_1_R agonist, and BQCA (benzyl quinolone carboxylic acid) derivatives, that are PAMs, were used as allosteric modulators (Table 7). It was found that BQCA-CCh bitopic compounds act as agonists. The highest potency and efficacy was observed for the compound containing BQCA moiety **81**. Comparing with reference compound **86**, which does not contain a CCh moiety but only the linker, revealed that the CCh moiety provides some of the agonist activity. In contrast, the TBPB-CCh bitopic ligand (**78**) showed partial agonism, while the reference **84** was a full agonist. The binding mode of **81** was investigated by docking to an active receptor model. The ammonium group of the CCh moiety forms a charge-assisted hydrogen bond with D105^3.32^, while the carbamate carbonyl group serves as a hydrogen bond acceptor for the hydroxyl group of Y408^7.43^. This is different from the carbachol binding mode, in which the carbamate structure has a different orientation. The BQCA moiety, located in the region of the extracellular loop, is stabilized by hydrophobic contacts with L174^ECL2^ and Y179^ECL2^ and a charge-assisted H-bond with K392^ECL3^. They concluded that partial agonism through bitopic compounds can be achieved not only by quenching orthosteric receptor activation by an allosteric moiety as in **81** but also by quenching bitopic activation of the receptor by an orthosteric moiety such as CCh in **78** (Schramm et al., 2019).

**TABLE 7:** Potency and efficacy induced by muscarinic agonists bitopic compounds HEK293t cells overexpressing the M1 receptor (Schramm et al., 2019).
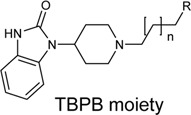
 
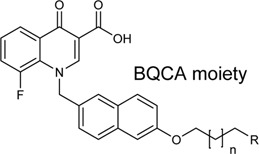

Cmpd	N	R	pEC_50_ nM ± SEM	% E_max_±SEM
**CCh**			6.97 ± 0.03	99 ± 1
**TBPB**			7.32 ± 0.02	83 ± 1
**BQCA**			7.20 ± 0.03	90 ± 1
**77 (TBPB)**	1	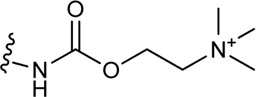	n.d.	n.d.
**78 (TBPB)**	3	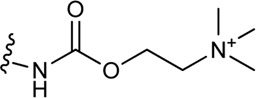	5.09 ± 0.24	12 ± 2
**79 (TBPB)**	6	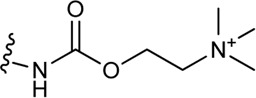	n.d.	n.d.
**80 (BQCA)**	1	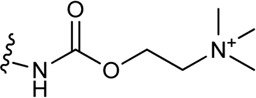	5.89 ± 0.01	66 ± 0.5
**81 (BQCA)**	3	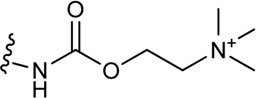	6.67 ± 0.02	78 ± 1
**82 (BQCA)**	6	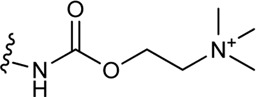	6.62 ± 0.03	28 ± 0.5
**83 (TBPB)**	1	H	6.05 ± 0.01	99 ± 1
**84 (TBPB)**	3	H	6.42 ± 0.01	97 ± 1
**85 (TBPB)**	6	H	7.38 ± 0.04	98 ± 2
**86 (BQCA)**	1	H	5.82 ± 0.02	35 ± 1
**87 (BQCA)**	3	H	n.d.	n.d.
**88 (BQCA)**	6	H	n.d.	n.d.

**FIGURE 7:**
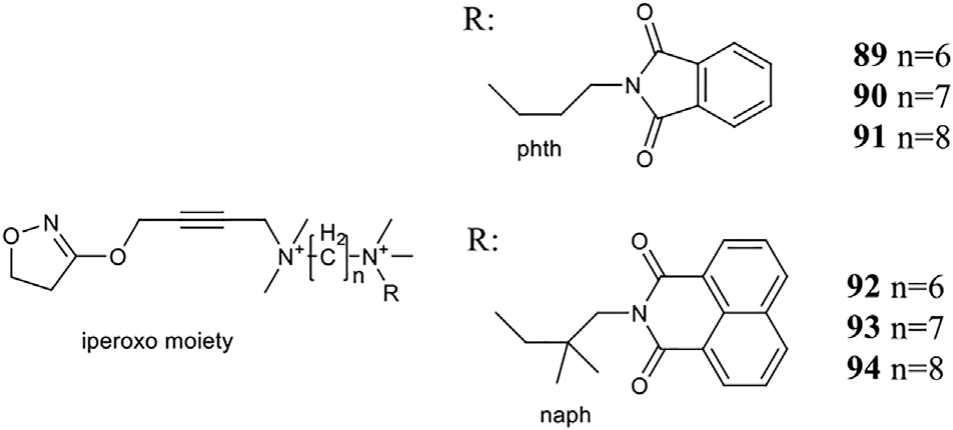
Iperoxo derivatives investigated at the M_1_ receptor in the study of Holze et al. (2020).

Holze et al. have shown that allosteric coupling of the M_1_R can induce conformational changes that affect intracellular signalling. They investigated two groups of M_1_R bitopic agonists and varied the length of the linker. Iperoxo, a known agonist, was selected as the PP motif, while two negative allosteric modulators, phtp (**89–91**) and naph (**92–94**), were incorporated as SP. (Figure 7) The latter differs from the phth derivative in two main respects: naph contains a larger and branched aliphatic linker. The two pharmacophores were linked by alkyl chains of different length (6–8C) (**89–94**). While the ligand affinities for the allosteric binding site were very similar within a ligand set, the ligand affinities for the orthosteric binding site depended on the length of the linker, where increasing linker length was correlated with increasing ligand affinity. From this information, it was concluded that the same binding mode was adopted by iperoxo in a series of bitopic compounds driven by its high affinity, and this was confirmed by MD simulations. Therefore, a series of bitopic ligands differing only in the length of the linker may be suitable to investigate the effect of allosteric coupling on signal transduction with subnanometer accuracy. Whereas the longest bitopic agonist, **91**, was able to stimulate all three G-protein families, **90** activated G_q/11_ and G_s_ proteins, **89** promoted signal transduction only via G_q/11_. **93** and **94** only activated G_q/11_ protein signalling, while **92** did not activate any signalling pathway, unlike **89**. None of the naph-based ligands were able to activate G_s_ and G_i/o_ signalling. These data suggest that different G-proteins show different sensitivities to M_1_R activation by these bitopic compounds. While G_q/11_ coupling is conserved in almost all bitopic ligands, G_s_ signalling is promoted only by two members of the phth series. G_i/o_ activation is particularly sensitive to the bitopic ligand structure with only **91** showing weak M_1_R/G_i/o_ coupling among the compounds tested. MD simulations show that binding of iperoxo results in a complete contraction of the extracellular parts of the ligand binding pocket. In contrast, the bitopic ligands of the phth series bind in such a way that they sterically inhibit the closure of the binding pocket. The extent of the conformational interference depends on the length of the linker and hence the position of the allosteric building block. Since the phth part of **89** is located close to the orthosteric binding site, it inhibits closure, resulting in a more open extracellular conformation. Elongation of the linker with additional methylene groups allowed for subnanometer regulation of the position of the allosteric building block, thereby progressively reducing the closure of the binding pocket, ultimately resulting in greater G-protein binding capacity. FRET measurements have demonstrated that the more closed ligand-binding pocket is associated with greater receptor conformational changes at the G-protein binding surface *via* an allosteric coupling mechanism. Consistent with this idea, **92**, a bitopic ligand with a branched and larger allosteric motif, did not induce conformational changes in M_1_R (Holze et al., 2020).

Wang et al. investigated two naltrexone derivatives substituted with isoquinoline at MOR. The isoquinoline moiety of these bitopic compounds is the SP that interacts with the allosteric site of MOR, and the epoxymorphinan moiety is the PP (Table 8). **NAQ** has a high affinity for MOR (K_i_ = 0.55 nM) and high selectivity for κ-opioid receptor (KOR) (48-fold) and δ-opioid receptor (DOR) (241-fold). Compared to DAMGO, it acts as a MOR antagonist in the ^35^S-GTP [γS]-binding assay with CHO cell lines expressing MOR. It showed less significant withdrawal effects compared to the well-known opioid antagonists naloxone and naltrexone. Similar properties were observed for the compound **NCQ** (K_i_ = 0.55, 40-fold KOR, 62-fold DOR selectivity), which shares the same PP part as **NAQ** and differs only in the SP. **NCQ** contains a methoxy at position 1 and a chloro functional group at position 4 of isoquinoline. However, in ^35^S-GTP (γS)-binding assay, **NCQ** behaved as a partial agonist. MD simulations and free energy calculations proposed that the allosteric part of **NAQ** and **NCQ** bind differently in the inactive structure and in the active structure, respectively. Docking studies have shown that the SP parts of **NAQ** and **NCQ** may occupy two different subdomains of the allosteric site of MOR, named ABD1 and ABD2. MD simulations were performed with three poses (**NAQ** inactive, **NCQ** active and inactive) obtained from the docking calculations and showed that the SP part of **NAQ** was bound to ABD1 in the inactive MOR. Although the SP motif occupied an allosteric site, no significant modulatory effect was observed on the binding of the PP, similar to the function of a silent allosteric modulator. In the inactive and active MOR the SP of **NCQ** showed positive allosteric modulation through binding to ABD2. Molecular modelling combined with interaction energy and distance analyses unravelled the molecular mechanisms of allosteric modulation of **NAQ** and **NCQ** and emphasized the importance of the chlorine and methoxy substituents of the isoquinoline ring for the allosteric modulatory function of **NCQ** (Wang et al., 2020).

**TABLE 8:** Binding affinities and functional efficacies of NAQ and NCQ (Wang et al., 2020).

Cmpd	Ki (nM±SEM)			MOR vs. KOR	MOR vs. DOR	MOR (^35^S) GTPγS binding	
	MOR	KOR	DOR			EC_50_ (nM ±SEM)	E_max_ of DAMGO % ± SEM
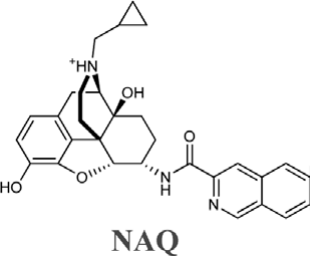	0.55 ± 0.15	26.45 ± 5.22	132.50 ± 27.01	48	241	4.36 ± 0.72	15.83 ± 2.53
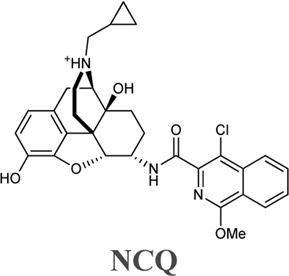	0.55 ± 0.01	22.20 ± 2.10	33.90 ± 0.50	40	62	1.74 ± 0.13	51.00 ± 0.40

### Binding Kinetics

Although ligand-receptor binding kinetics might have a fundamental role in the development of drug candidates, it is still often overlooked in the early phase of drug discovery. In line with the increased interest in the field, more and more kinetics data (among others association and dissociation rate, residence time, etc.) have been published in the literature, however the magnitude still lags behind the amount of affinity and selectivity data available especially regarding only the allosteric and bitopic ligands. Furthermore, the interpretation of the kinetic data might be hindered by the probe dependence as observed in a prototypical competitive radioligand binding assay for H_1_ receptor antagonists, although that aspect is often not considered (Bosma et al., 2019). In line with the relatively limited amount of recent papers, first we refer the readers to recent general review articles on binding kinetics (Sykes et al., 2019; Hoare et al., 2020; Rafael et al., 2020; van der Velden et al., 2020). Very recently a book chapter collecting available kinetic data of GPCR ligands together with experimental evidence for properties that influence the residence time were published (Potterton et al., 2022). The repository enables researchers to analyse the relationship between the structure and the kinetic parameters as well as provides data for the development of predictive algorithms. The authors also outline machine learning workflows to predict residence time. Sykes et al. reviewed recently the literature related to the binding kinetics of GPCR ligands (Sykes et al., 2019). They discussed the theoretical aspects, the experimental methods and their limitations, detailed several factors influencing binding kinetics among others they explored the role of allosteric modulators, that by definition act through the modulation of the binding kinetics of the endogenous or orthosteric ligands. The authors also discuss some molecular level features including shielding the hydrogen bonds from water that affects the binding kinetics.

Although shielding the hydrogen bonds was thought to decrease residence time, in a recent case study on CCR2 receptor, MD simulations of Magarkar et al. suggested that even shielding an intra protein hydrogen bond can enhance the residence time of ligands through the preservation of the binding site rigidity (Magarkar et al., 2019). The ECL2 loop, that is regularly engaged with bitopic compounds, was also proposed to modulate the binding kinetics (Sykes et al., 2019; van der Velden et al., 2020). Already one of the seminal works in the field of modelling the binding pathway to GPCRs, which investigated the binding of three antagonists and an agonists to the β2-adrenoreceptor and one agonist to the β1-adrenoreceptor with MD simulations, highlighted the role of the ECL2 loop and the extracellular vestibule. Interestingly, even the highest barrier of binding often corresponds to the association with the extracellular vestibule even though the binding requires conformational change of the receptor and the ligand has to enter through a narrow passage (Dror et al., 2011). In several receptors, ECL2 were proposed to function as a lid facilitating the entrance and exit of the ligands (Thomas et al., 2016; Wacker et al., 2017; Frank et al., 2018). One of these studies investigated the binding kinetics of cariprazine and aripiprazole. As a prototypical bitopic compounds we exemplify here the effect of the SBP on the binding kinetics through them (Frank et al., 2018). At the D_3_ receptor, aripiprazole exhibits a slow monophasic dissociation, while cariprazine displays a rapid biphasic behaviour. Interestingly, in the D_2_ receptor both compounds display a slow dissociation. These differences may influence the *in vivo* action of the drugs. Interactions with ECL2 residues influence the residence time in other receptors like in the β_2_ and A_2A_ receptors, as well (Guo et al., 2016; Masureel et al., 2018). Gaussian accelerated molecular dynamics revealed the role of the ECL2 loop in the formation of allosteric sites for PAMs in the adenosine A_1_ receptor (Miao et al., 2018) and unveiled an intermediate binding site between ECL2 and TM1 for caffein in the adenosine A_2A_ receptor. The authors analysed the effect of more general features like physicochemical properties of the ligand (e.g., lipophilicity) and close contact residue numbers on the drug-receptor dissociation.

Van der Velden et al. summarized structural considerations in relation to binding kinetics presenting the results through four case studies (van der Velden et al., 2020). They showcased the role of the ECL2 loop in the regulation of the ligand kinetics through tiotropium binding to the M_3_R and M_2_R receptors (Kruse et al., 2012; Tautermann et al., 2013). The more open, flexible ECL2 loop conformation was linked to the shorter residence time observed in the M_2_R receptor. Through the example of ZM241385, an A_2A_ receptor antagonists they highlighted the role of molecular dynamics and mutation experiments in providing structural background for observed kinetics behaviour (Guo et al., 2016). Another example was focused on the β_2_ adrenoreceptor. Salmeterol, a bitopic compound displays a 5–7 fold higher residence time compared to salbutamol and epinephrin, both binding only to the orthosteric site (Figure 3C). As salmoterol and salbutamol share the orthosteric binding motif, the interactions in the extracellular site are linked to the increased residence time (Masureel et al., 2018). They also discussed other aspects, like the effect of natural receptor variants, ligand variants and probe dependency.

Riddy et al. investigated the binding kinetics of H_3_ receptor antagonists/inverse agonists (Riddy et al., 2019). Although the binding mode of the compounds were not investigated experimentally, they likely form interactions outside the orthosteric pocket, too therefore can be considered bitopic. The different pharmacological profile and the residence time of the compounds might be linked to their preclinical and clinical efficacy. Furthermore, H_3_ and off-target sigma-1 receptor occupancy may contribute to paradoxical efficacy of some compounds. In the study of Pedersen et al. (2020) the differential binding kinetics profile of the agonists were not linked to the functional bias, as the bias profile of the selected agonists were not time-dependent and despite the difference in their binding kinetic properties they can display the same degree of bias.

Bitopic compounds and allosteric modulators may directly bind to the secondary binding pocket, however, during the association and dissociation process the secondary site plays a crucial role for the appropriate positioning of all compounds. While experiments rarely shed light on the structural details of binding, molecular dynamics simulations can explore the atomistic process and are useful to predict residence time (Potterton et al., 2019; Decherchi and Cavalli, 2020; Lamim Ribeiro et al., 2020; Salmaso and Jacobson, 2020; Bekker et al., 2021; Kokh and Wade, 2021). Ribeiro and co-workers recently used machine learning and infrequent metadynamics to efficiently predict kinetic rates, transient conformational states, and molecular determinants of drug dissociation on the MOR (Lamim Ribeiro et al., 2020). While both investigated compounds bind to the orthosteric pocket, the transient conformational state for the dissociation was identified around the secondary binding pocket suggesting a key role of the secondary site in the association/dissociation process. In dynamic docking simulations Bekker et al. investigated β_2_-adrenoreceptor antagonists identifying several stable and metastable conformational states for the compounds along their association/dissociation path (Bekker et al., 2021). Based on these simulations they propose a way to develop allosteric modulators to inhibit the receptor by blocking the path of the endogenous ligand to the orthosteric site. Metastable binding sites play a crucial role in the study of Gaiser et al. as well (Gaiser et al., 2019). They developed homobivalent bitopic ligands for β_2_AR to target the OBP and a previously identified metastable binding site as an allosteric site. Among others the residence time of orthosteric and bitopic A_2A_ receptor binders was predicted with ensemble based steered molecular dynamics (Potterton et al., 2019). Analysis of the pathways revealed dominant interactions, residues influencing the dissociation time and the calculations proposed that changes in water-ligand energy from the ligand in the binding pocket to the extracellular vestibule was the main factor in the determination of residence time. While hydrophilic ligands are expected to access the orthosteric binding site, that is deeply embedded in the center of the receptor, from the aqueous phase, hydrophobic compounds were proposed to entry through lipid pathways. The examples detailed in this part explore the traditional pathway, however cholesterol and other ligands might enter the receptor from the membrane. As an exciting study we refer to the work of Guixá-González et al. who investigated the cholesterol access to the A_2A_R with combined computational and experimental methods. They showed that cholesterol’s impact on A_2A_R-binding affinity goes beyond pure allosteric modulation and unveils a new interaction mode between cholesterol and the A2AR (Guixà-González et al., 2017). Similar findings were collected and analysed in a recent review dedicated to the role of the lipid bilayer in the binding of the ligands to the orthosteric and allosteric sites (Szlenk et al., 2019). Even though in this review we focused mainly on the secondary binding pocket in the extracellular vestibule that is accessible through the aqueous phase, some allosteric sites on the receptor surface can only be targeted through the membrane fortifying that investigation of the binding pathways through the membrane is also crucial.

## Design Approaches for Allosteric and Bitopic Compounds

During the previous sections we often pointed out the value of computational approaches in the investigation of both allosteric and bitopic compounds. Due to the tremendous number of studies a comprehensive overview of the computational approaches to design allosteric (Wold et al., 2019; Chatzigoulas and Cournia, 2021) and bitopic ligands (Newman et al., 20162020; Fronik et al., 2017) for GPCRs warrant a separate review (Basith et al., 2018; Raschka and Kaufman, 2020; Ballante et al., 2021), we could only highlight here a few important studies to draw attention towards their usefulness in drug discovery settings (Dehua Yang et al., 2021).

Allosteric sites are less conserved and therefore they can be exploited to design ligands with high selectivity and modalities that could not be achieved from the orthosteric site. The increasing number of experimental GPCR structures urges the use of structure-based methods. However, the identification of the allosteric sites remains challenging as they often form fully only in the presence of an allosteric ligand following an induced fit mechanism. Nevertheless, several computational approach were developed to facilitate the spotting of new allosteric sites like Allosite (Huang et al., 2013), AlloFinder (Huang et al., 2018), ExProSE (Greener et al., 2017), Fpocket (Le Guilloux et al., 2009; Schmidtke et al., 2010), FTmap (Brenke et al., 2009; Kozakov et al., 2015), GRID (Goodford, 1985), LIGSITE^csc^ (Huang and Schroeder, 2006), SiteMap (Halgren, 2009) and MixMD (Ghanakota and Carlson, 2016). FTMap and FTSite was recently shown to perform well on identifying GPCR allosteric sites with limitations on those occurring on the protein-membrane interface that could be attributed to the development of the program originally for soluble globular proteins (Wakefield et al., 2019).

Even after the identification of the allosteric site, simple docking might not always be successful due to induced fit binding. Furthermore, allosteric modulators are prone to “steep” SAR, obscure relationship between the binding affinity and functional effect and slow kinetics (on and/or off rates) that hinders their discovery and design (Congreve et al., 2017b). Huang et al. developed a protocol combining homology modelling and docking to find novel allosteric modulators of the orphan GPR68 and GPR65 receptors (Huang et al., 2015). They generated over three thousand homology models and docked their experimentally validated active compound lorazepam and decoy compounds to identify putative binding sites. They optimized the binding site around the bound ligand and redocked the ligand and the decoys again until a stable docking mode emerged. That plausible binding site was utilized to dock over 3.1 million lead-like compounds. From the selected 17 hits four increased cAMP production. Docking close analogues of the hit compounds lead to another 25 compounds for testing among them 13 with higher activity than the reference compound lorazepam. Similar protocol was utilized for the GPR65 receptor as well showing that the protocol might be applied to a broader field. While this protocol might be applied to several—even orphan—GPCRs it requires at least one experimentally determined known active compound that might be hard to get for other orphan GPCRs and close enough homology to templates that warrant the homology modelling. Nevertheless, this is a great example how the combined experimental and computational approaches can lead to the identification of novel allosteric modulators even for orphan GPCR targets. Miao et al. focused on the identification of novel, chemically diverse allosteric modulators of the M_2_ receptor (Miao et al., 2016). The authors used accelerated molecular dynamics to account for receptor flexibility and to generate an ensemble of structures for docking. After retrospective validation virtual screening coupled with induced fit docking (IFD) was applied to select compounds targeting the IXO-nanobody-bound active and the QNB-bound inactive M_2_ mAChR for testing. The method successfully identified both positive and negative allosteric modulators and clearly demonstrate that accounting for receptor flexibility is a key in the discovery of allosteric modulators. Nevertheless, for less flexible binding sites even simple docking protocols might be plausible as demonstrated by Korczynska et al. identifying a positive allosteric modulator that potentiates antagonist binding leading to subtype selectivity at the M_2_ muscarinic acetylcholine receptor (Korczynska et al., 2018). Since allosteric modulators are often small and rigid compounds, fragment based approaches (Keserű et al., 2016) emerge as a plausible choice for the design that is supported by several successful application (Christopher et al., 2015; Orgován et al., 2019). Furthermore, covalent approaches should not be overlooked either to aid structurally informed rational design (Lu and Zhang, 2017; Bian et al., 2020; Wenchao Lu et al., 2021).

Bitopic compounds are in the forefront of drug development for GPCRs as they can combine the advantages of targeting the orthosteric and a secondary site (Newman et al., 20162020; Fronik et al., 2017). Fragment based methods are often applied to design novel bitopic compounds (Vass et al., 2014; Egyed et al., 2021). Recently our group have developed a computational protocol to design specific, selective receptor ligands (Egyed et al., 2021). First fragments were docked to the orthosteric binding site of the receptors available in experimental structures (D_3_: PDB ID: 3PBL (Chien et al., 2010), 5-HT_1B_: PDB ID: 4IAQ (Wang et al., 2013), 4IAR (Wang et al., 2013); 5-HT_2B_: PDB ID: 4IB4 (Wacker et al., 2013), 4MC3 (Liu et al., 2013); H_1_: PDB ID 3RZE (Shimamura et al., 2011) and M_1_: PDB ID: 5CXV (Thal et al., 2016)), or a homology model in case of the D_2_ receptor. Then, virtual fragment screening was performed against the secondary binding site of the combined protein-ligand complex. The identified SBP fragment was then linked to the OBP core by a linker. As a control, the resulting bitopic compounds were docked back into the initial crystal structure. This protocol has been validated by designing selective D_2_/D_3_, 5-HT_1B_/5-HT_2B_ and H_1_/M_1_ receptors. Docking-based fragment evolution approach utilizes the same methodology as exemplified on the design of β_1_ and β_2_ receptor bitopic compounds (Chevillard et al., 2021). The fragment evolution protocol merges fragment growing with a matrix-based strategy that was originally implemented for potency optimization (Chevillard et al., 2019). First, possible OBP fragments were docked and they were evaluated using the concept of ligand efficiency. Next, fragment growing surrogates suitable for reactive alkylation were defined and docked to the secondary binding pocket. Surrogates that overlap with the core OBP fragment or was marked favourably in both receptors were removed from the top ranked compounds, the remaining top surrogates were kept for further investigation. The OBP fragments were reacted *in silico* with the surrogates, the resulting compounds were docked into the receptors to ensure pose fidelity. Based on these calculations the best surrogates were selected as secondary binding motif for the β_1_ and β_2_ receptor, respectively. The approach was validated by the synthesis and experimental evaluation of the designed compounds. Classical docking and virtual screening approaches could be also utilized for the development of bitopic compounds (Cao et al., 2018) and even to develop fluorescent GPCR probes (Prokop et al., 2021). We highlight here a study that utilized structure guided design of GPCR polypharmacology (Kampen et al., 2021). Kampen et al. aimed to design dual A_2A_/D_2_ bitopic compounds that was very challenging due to the significantly different binding sites of the receptors. First, docking based structural analysis confirmed that dual-target ligands of the A_2A_R and D_2_R could be obtained by targeting the orthosteric and secondary pockets. Then, they designed potential dual targeting virtual chemical libraries that could be rapidly synthesized. The prepared libraries were screened virtually with docking on the A_2A_ and the D_2_ receptor to select hits. From them one promising compound was selected and developed further with SAR investigations.

Discussing the recent advances in the allosteric and bitopic field we pointed out several times the usefulness of MD based methods. These simulations can explore the differences in the interaction patterns of congeneric molecules more sensitively compared to docking that could be important to understand the different functional outcome of these ligands (Egyed et al., 2020) and to design compounds with specific pharmacological profile (McCorvy et al., 2018) and they might reveal cryptic pockets opened by ligands (Ferruz et al., 2018) that might be overlooked in simple docking calculations. Mutation studies combined with extensive molecular dynamics modelling the dissociation of the ligands was utilized to clarify the structural basis of the long duration of action and kinetic selectivity of tiotropium for the M_3_ receptor (Tautermann et al., 2013). A similar study aimed to clarify the molecular determinants of the bitopic binding mode of a negative allosteric modulator of the dopamine D_2_ receptor (Draper-Joyce et al., 2018). MDs combined with docking linked the degree of closure of the extracellular loop region to the extent of ligand bias and highlighted the importance of the appropriate receptor conformation for virtual screening at the 5-HT_2B_ receptor (Denzinger et al., 2020). A similar concept was presented by Bermudez et al. proposing that agonists with extended binding modes selectively interfere with binding pocket closure and through divergent allosteric coupling that leads to ligand bias (Bermudez and Bock, 2019).

The structure-based methods clearly benefit from the increase of published GPCR structures, especially that more and more active structures are available, however the design still remains challenging. Nevertheless, with more template available for homology modelling and the publication of AlphaFold (Jumper et al., 2021) facilitate the structure-based methods for targets previously out of scope for these methods broadening the applicability spectrum. While we mainly highlighted structure based approaches classical ligand based methods and cheminformatics also contribute to the development of bitopic GPCR ligands (Basith et al., 2018; James and Heifetz, 2018; Raschka and Kaufman, 2020).

## Conclusion

GPCRs are one of the largest families of receptors and are among the most targeted proteins for drug discovery. One of the major challenges in the field is the identification of subtype and functionally selective compounds with high potency, designed efficacy and appropriate binding kinetics profile, which are essential to avoid side effects. The secondary binding pocket plays a prominent role in achieving selectivity, while orthosteric ligands are mainly responsible for affinity and functional activity. Bitopic compounds combine the properties of orthosteric and allosteric pharmacophores. With the continuous expansion of available GPCR structures, the secondary binding sites of the receptors are becoming better understood, allowing the construction of complex ligands with designed pharmacological profile. In this review, we have provided an insight into allosteric modulators of class A GPCRs and a detailed review of bitopic compounds that have been released in the last years. We have highlighted the influence of the secondary site in affinity, selectivity, functional selectivity and binding kinetics. The increasing amount of pharmacological data and new structures together with appropriate modelling tools can contribute to the design of allosteric and bitopic drug candidates with an optimized pharmacology profile and thus accelerating the drug discovery against diseases with high unmet medical need.
