# Supplementary material for: The Impact of the Secondary Binding Pocket on the Pharmacology of Class A GPCRs
Source: Front Pharmacol. 2022 Mar 9;13:847788. doi: 10.3389/fphar.2022.847788 (PMC8959758; doi:10.3389/fphar.2022.847788)
Supplement: Supplementary file 1 [file DataSheet1.DOCX]

Supplementary Material

**Table of contents**

[1 Table S1. GPCR structures with allosteric ligand 1](#_Toc94254950)

[2 Table S2. Active aminerg GPCR structures 9](#_Toc94254951)

[3 Table S3. D_3_ selective SAR study from PCMPA, bindig affinity data^1^ 14](#_Toc94254952)

[4 Table S4. SAR study from PD-128907 and PF-592379, binding affinity data^2^ 18](#_Toc94254953)

[5 Table S5. Binding data for 3-thiophenephenyl and 4-thiazolylphenyl fluoridesubstituted N-phenylpiperazine analogs^5^ 20](#_Toc94254954)

[References 22](#_Toc94254955)

# Table S1. GPCR structures with allosteric ligand

| **IUPHAR** | **PDB** | **Class** | **State** | **Ligand** | **Function** | **Reference** | **PDB Date** |
| --- | --- | --- | --- | --- | --- | --- | --- |
|  |  |  |  |  |  |  |  |
| M1 | 6WJC | A (Rhodopsin) | Inactive | Hyoscyamine | Antagonist | [10.1126/SCIENCE.AAX2517](https://dx.doi.org/10.1126/SCIENCE.AAX2517) | 2020.07.08 |
|  |  |  |  | Muscarinic toxin 7 | NAM |  |  |
| M2 | 6OIK | A (Rhodopsin) | Active | Iperoxo | Agonist | [10.1126/SCIENCE.AAW5188](https://dx.doi.org/10.1126/SCIENCE.AAW5188) | 2019.05.08 |
|  |  |  |  | 2CU | PAM |  |  |
| M2 | 4MQT | A (Rhodopsin) | Active | Iperoxo | Agonist | [10.1038/NATURE12735](https://dx.doi.org/10.1038/NATURE12735) | 2013.11.27 |
|  |  |  |  | 2CU | PAM |  |  |
| A1 | 7LD3 | A (Rhodopsin) | Active | adenosine {2-Amino-4-[3,5-bis(trifluoromethyl)phenyl]thiophen-3-yl}(4-chlorophenyl)methanone | Agonist PAM | [10.1038/S41586-021-03897-2](https://dx.doi.org/10.1038/S41586-021-03897-2) | 2021.09.08 |
|  |  |  |  |  |  |  |  |
| β 2 | 6OBA | A (Rhodopsin) | Inactive | (S)-Alprenolol | Antagonist NAM | [10.1038/S41589-020-0549-2](https://dx.doi.org/10.1038/S41589-020-0549-2) | 2020.03.25 |
|  |  |  |  | 6-bromo-N~2~-phenylquinazoline-2,4-diamine | NAM |  |  |
| β 2 | 6N48 | A (Rhodopsin) | Active | BI-167107 | Agonist | [10.1126/SCIENCE.AAW8981](https://dx.doi.org/10.1126/SCIENCE.AAW8981) | 2019.06.26 |
|  |  |  |  | 2-[[(3R)-4-(4-Tert-butylphenyl)-3-[[2-(4-methoxyphenyl)sulfanyl-5-[methyl(propan-2-yl)sulfamoyl]benzoyl]amino]butanoyl]amino]acetic acid | PAM |  |  |
| GPBA | 7CFN | A (Rhodopsin) | Active | INT-777 | Agonist | [10.1038/S41586-020-2569-1](https://dx.doi.org/10.1038/S41586-020-2569-1) | 2020.09.09 |
|  |  |  |  | INT-777 | PAM |  |  |
| CaS | 7E6T | C (Glutamate) | Inactive | Calcium ion | Agonist | [10.7554/ELIFE.68578](https://dx.doi.org/10.7554/ELIFE.68578) | 2021.09.22 |
|  |  |  |  | Cyclomethyltryptophan | PAM |  |  |
| CaS | 7M3J | C (Glutamate) | Inactive | NPS-2143 | NAM | [10.1038/S41586-021-03691-0](https://dx.doi.org/10.1038/S41586-021-03691-0) | 2021.06.30 |
| CaS | 7DD6 | C (Glutamate) | Active | Calcium ion | Agonist | [10.1126/SCIADV.ABG1483](https://dx.doi.org/10.1126/SCIADV.ABG1483) | 2021.06.16 |
|  |  |  |  | Tryptophan | PAM |  |  |
| CaS | 7DTU | C (Glutamate) | Inactive | Tryptophan | PAM | [10.1038/S41422-021-00474-0](https://dx.doi.org/10.1038/S41422-021-00474-0) | 2021.03.10 |
| CaS | 7DTV | C (Glutamate) | Inactive | Calcium ion | Agonist | [10.1038/S41422-021-00474-0](https://dx.doi.org/10.1038/S41422-021-00474-0) | 2021.03.10 |
|  |  |  |  | Tryptophan | PAM |  |  |
| CB1 | 6KQI | A (Rhodopsin) | Inactive | CP55940 | Agonist | [10.1038/S41589-019-0387-2](https://dx.doi.org/10.1038/S41589-019-0387-2) | 2019.10.23 |
|  |  |  |  | ORG27569 | NAM |  |  |
| CXCR2 | 6LFL | A (Rhodopsin) | Inactive | 4-[[3,4-Dioxo-2-[[(1R)-1-(4-propan-2-ylfuran-2-yl)propyl]amino]cyclobuten-1-yl]amino]-3-hydroxy-N,N-dimethylpyridine-2-carboxamide | Allosteric antagonist | [10.1038/S41586-020-2492-5](https://dx.doi.org/10.1038/S41586-020-2492-5) | 2020.09.02 |
| CCR7 | 6QZH | A (Rhodopsin) | Inactive | SCHEMBL13407377 | Allosteric antagonist | [10.1016/J.CELL.2019.07.028](https://dx.doi.org/10.1016/J.CELL.2019.07.028) | 2019.09.04 |
| CCR9 | 5LWE | A (Rhodopsin) | Inactive | Vercirnon | Allosteric antagonist | [10.1038/NATURE20606](https://dx.doi.org/10.1038/NATURE20606) | 2016.12.07 |
| C5a1 | 6C1Q | A (Rhodopsin) | Inactive | PMX53 | Antagonist | [10.1038/S41594-018-0067-Z](https://dx.doi.org/10.1038/S41594-018-0067-Z) | 2018.05.30 |
|  |  |  |  | NDT 9513727 | NAM |  |  |
| C5a1 | 6C1R | A (Rhodopsin) | Inactive | PMX53 | Antagonist | [10.1038/S41594-018-0067-Z](https://dx.doi.org/10.1038/S41594-018-0067-Z) | 2018.05.30 |
|  |  |  |  | Avacopan | NAM |  |  |
| D1 | 7CKZ | A (Rhodopsin) | Active | dopamine | Agonist | [10.1016/J.CELL.2021.01.028](https://dx.doi.org/10.1016/J.CELL.2021.01.028) | 2021.03.03 |
|  |  |  |  | Mevidalen | PAM |  |  |
| D1 | 7LJC | A (Rhodopsin) | Active | 743408-71-1 | Agonist | [10.1101/2021.02.07.430101](https://dx.doi.org/10.1101/2021.02.07.430101) | 2021.03.03 |
|  |  |  |  | Mevidalen | PAM |  |  |
| D1 | 7LJD | A (Rhodopsin) | Active | dopamine | Agonist | [10.1101/2021.02.07.430101](https://dx.doi.org/10.1101/2021.02.07.430101) | 2021.03.03 |
|  |  |  |  | Mevidalen | PAM |  |  |
| FFA1 | 5KW2 | A (Rhodopsin) | Intermediate | (3~{S})-3-cyclopropyl-3-[2-[1-[2-[2,2-dimethylpropyl-(6-methylpyridin-2-yl)carbamoyl]-5-methoxy-phenyl]piperidin-4-yl]-1-benzofuran-6-yl]propanoic acid | Allosteric agonist | [10.1038/S41467-017-01240-W](https://dx.doi.org/10.1038/S41467-017-01240-W) | 2018.05.02 |
| FFA1 | 5TZY | A (Rhodopsin) | Intermediate | MK-8666 | Agonist | [10.1038/NSMB.3417](https://dx.doi.org/10.1038/NSMB.3417) | 2017.06.07 |
|  |  |  |  | (2S,3R)-3-Cyclopropyl-3-[(2R)-2-[1-[(1S)-1-[5-fluoro-2-(trifluoromethoxy)phenyl]ethyl]piperidin-4-yl]-3,4-dihydro-2H-chromen-7-yl]-2-methylpropanoic acid | PAM |  |  |
| SMO | 6XBL | F (Frizzled) | Active | Cholesterol | Agonist | [10.1038/S41589-020-0646-2](https://dx.doi.org/10.1038/S41589-020-0646-2) | 2020.09.30 |
|  |  |  |  | SAG | PAM |  |  |
| SMO | 6XBM | F (Frizzled) | Active | 24,25-Epoxy-cholesterol | Agonist | [10.1038/S41589-020-0646-2](https://dx.doi.org/10.1038/S41589-020-0646-2) | 2020.09.30 |
|  |  |  |  | 24,25-Epoxy-cholesterol | PAM |  |  |
| GABAB2 | 7EB2 | C (Glutamate) | Active | Baclofen | Agonist | [10.1038/S41586-021-03507-1](https://dx.doi.org/10.1038/S41586-021-03507-1) | 2021.05.05 |
|  |  |  |  | BHFF | PAM |  |  |
| GABAB2 | 7CA3 | C (Glutamate) | Active | BHFF | PAM | [10.1016/J.JMB.2020.09.023](https://dx.doi.org/10.1016/J.JMB.2020.09.023) | 2020.11.11 |
| GABAB2 | 7CUM | C (Glutamate) | Inactive | CGP54626 | Antagonist | [10.1016/J.JMB.2020.09.023](https://dx.doi.org/10.1016/J.JMB.2020.09.023) | 2020.11.11 |
|  |  |  |  | Unknown ligand | NAM |  |  |
| GABAB1 | 6W2Y | C (Glutamate) | Inactive | CGP55845 | Inverse agonist | [10.1038/S41586-020-2469-4](https://dx.doi.org/10.1038/S41586-020-2469-4) | 2020.07.01 |
|  |  |  |  | 1-Stearoyl-2-oleoyl-sn-glycero-3-phosphoethanolamine | NAM |  |  |
| GABAB2 | 6W2X | C (Glutamate) | Inactive | CGP55845 | Inverse agonist | [10.1038/S41586-020-2469-4](https://dx.doi.org/10.1038/S41586-020-2469-4) | 2020.07.01 |
|  |  |  |  | 1-Stearoyl-2-oleoyl-sn-glycero-3-phosphoethanolamine | NAM |  |  |
| GABAB2 | 7C7Q | C (Glutamate) | Active | Baclofen | Agonist | [10.1038/S41422-020-0350-5](https://dx.doi.org/10.1038/S41422-020-0350-5) | 2020.07.01 |
|  |  |  |  | BHFF | PAM |  |  |
| GABAB2 | 6WIV | C (Glutamate) | Inactive | [(2R)-3-[(Z)-icos-11-enoyl]oxy-2-[(Z)-octadec-9-enoyl]oxypropyl] 2-(trimethylazaniumyl)ethylphosphate | NAM | [10.1038/S41586-020-2452-0](https://dx.doi.org/10.1038/S41586-020-2452-0) | 2020.07.01 |
|  |  |  |  | (2R)-3-{[(S)-(2-aminoethoxy)(hydroxy)phosphoryl]oxy}-2-{[(9Z)-octadec-9-enoyl]oxy}propyl(5Z,8Z,11Z,14Z)-icosa-5,8,11,14-tetraenoate |  |  |  |
| GABAB2 | 6UO8 | C (Glutamate) | Active | (R)-(3-aminopropyl)methylphosphinic acid | Agonist | [10.1038/S41586-020-2408-4](https://dx.doi.org/10.1038/S41586-020-2408-4) | 2020.06.10 |
|  |  |  |  | N~4~,N~6~-dicyclopentyl-2-(methylsulfanyl)-5-nitropyrimidine-4,6-diamine | PAM |  |  |
| GLP-1 | 7EVM | B1 (Secretin) | Active | N-Tert-butyl-6,7-dichloroquinoxalin-2-amine | Ago-PAM | [10.1038/S41467-021-24058-Z](https://dx.doi.org/10.1038/S41467-021-24058-Z) | 2021.08.11 |
| GLP-1 | 7DUR | B1 (Secretin) | Active | N-Tert-butyl-6,7-dichloroquinoxalin-2-amine | Ago-PAM | [10.1038/S41467-021-24058-Z](https://dx.doi.org/10.1038/S41467-021-24058-Z) | 2021.08.11 |
| GLP-1 | 6VCB | B1 (Secretin) | Active | GLP-1 | Agonist | [10.1038/S41589-020-0589-7](https://dx.doi.org/10.1038/S41589-020-0589-7) | 2020.07.22 |
|  |  |  |  | LSN3160440 | PAM |  |  |
| GLP-1 | 6LN2 | B1 (Secretin) | Inactive | PF-06372222 | NAM | [10.1038/S41467-020-14934-5](https://dx.doi.org/10.1038/S41467-020-14934-5) | 2020.03.18 |
|  |  |  |  |  |  |  |  |
| GLP-1 | 6KK1 | B1 (Secretin) | Inactive | PF-06372222 | NAM | [10.1107/S2052252519013496](https://dx.doi.org/10.1107/S2052252519013496) | 2019.11.13 |
| GLP-1 | 6KJV | B1 (Secretin) | Inactive | PF-06372222 | NAM | [10.1107/S2052252519013496](https://dx.doi.org/10.1107/S2052252519013496) | 2019.11.13 |
| GLP-1 | 6KK7 | B1 (Secretin) | Inactive | PF-06372222 | NAM | [10.1107/S2052252519013496](https://dx.doi.org/10.1107/S2052252519013496) | 2019.11.13 |
| GLP-1 | 5VEW | B1 (Secretin) | Inactive | PF-06372222 | NAM | [10.1038/NATURE22378](https://dx.doi.org/10.1038/NATURE22378) | 2017.05.24 |
| glucagon | 5XEZ | B1 (Secretin) | Inactive | NNC0640 | NAM | [10.1038/NATURE22363](https://dx.doi.org/10.1038/NATURE22363) | 2017.05.24 |
| glucagon | 5XF1 | B1 (Secretin) | Inactive | NNC0640 | NAM | [10.1038/NATURE22363](https://dx.doi.org/10.1038/NATURE22363) | 2017.05.24 |
| GLP-1 | 5VEX | B1 (Secretin) | Inactive | NNC0640 | NAM | [10.1038/NATURE22378](https://dx.doi.org/10.1038/NATURE22378) | 2017.05.17 |
| glucagon | 5EE7 | B1 (Secretin) | Inactive | Octaethylene glycol monoethyl ether | Antagonist | [10.1038/NATURE17414](https://dx.doi.org/10.1038/NATURE17414) | 2016.04.20 |
|  |  |  |  | MK-0893 | NAM |  |  |
| mGlu5 | 7P2L | C (Glutamate) | Inactive | Alloswitch-1 | NAM | [10.1016/J.CELREP.2021.109648](https://dx.doi.org/10.1016/J.CELREP.2021.109648) | 2021.09.08 |
| mGlu2 | 7EPE | C (Glutamate) | Inactive | 4-(1-Methylpyrazol-4-yl)-7-[[(2~{S})-2-(trifluoromethyl)morpholin-4-yl]methyl]quinoline-2-carboxamide | NAM | [10.1038/S41586-021-03641-W](https://dx.doi.org/10.1038/S41586-021-03641-W) | 2021.06.23 |
| mGlu2 | 7EPF | C (Glutamate) | Inactive | (8~{R})-4-[2,4-Bis(fluoranyl)phenyl]-8-methyl-7-[(2-methylpyrazol-3-yl)methyl]-6,8-dihydro-5~{H}-1,7-naphthyridine-2-carboxamide | NAM | [10.1038/S41586-021-03641-W](https://dx.doi.org/10.1038/S41586-021-03641-W) | 2021.06.23 |
| mGlu2 | 7E9G | C (Glutamate) | Active | Eglumetad | Agonist | [10.1038/S41586-021-03495-2](https://dx.doi.org/10.1038/S41586-021-03495-2) | 2021.06.23 |
|  |  |  |  | 1-butyl-3-chloranyl-4-(4-phenylpiperidin-1-yl)pyridin-2-one | PAM |  |  |
| mGlu5 | 6FFI | C (Glutamate) | Inactive | 2-[2-(3-methoxyphenyl)ethynyl]-6-methyl-pyridine | NAM | [10.1021/ACS.JMEDCHEM.7B01722](https://dx.doi.org/10.1021/ACS.JMEDCHEM.7B01722) | 2018.03.07 |
| mGlu5 | 6FFH | C (Glutamate) | Inactive | Fenobam | NAM | [10.1021/ACS.JMEDCHEM.7B01722](https://dx.doi.org/10.1021/ACS.JMEDCHEM.7B01722) | 2018.03.07 |
| mGlu5 | 5CGD | C (Glutamate) | Inactive | Q27455393 | NAM | [10.1021/ACS.JMEDCHEM.5B00892](https://dx.doi.org/10.1021/ACS.JMEDCHEM.5B00892) | 2015.08.12 |
| mGlu5 | 5CGC | C (Glutamate) | Inactive | Q27455391 | NAM | [10.1021/ACS.JMEDCHEM.5B00892](https://dx.doi.org/10.1021/ACS.JMEDCHEM.5B00892) | 2015.08.12 |
| mGlu5 | 4OO9 | C (Glutamate) | Inactive | Mavoglurant | NAM | [10.1038/NATURE13396](https://dx.doi.org/10.1038/NATURE13396) | 2014.07.02 |
| mGlu1 | 4OR2 | C (Glutamate) | Inactive | FITM | NAM | [10.1126/SCIENCE.1249489](https://dx.doi.org/10.1126/SCIENCE.1249489) | 2014.03.19 |
| P2Y1 | 4XNV | A (Rhodopsin) | Intermediate | BPTU | NAM | [10.1038/NATURE14287](https://dx.doi.org/10.1038/NATURE14287) | 2015.04.01 |
| PAR2 | 5NDZ | A (Rhodopsin) | Intermediate | AZ3451 | Allosteric antagonist | [10.1038/NATURE22309](https://dx.doi.org/10.1038/NATURE22309) | 2017.05.03 |

# Table S2. Active aminerg GPCR structures

| **IUPHAR** | **PDB** | **Cl.** | **State** | **Ligand** | **Function** | **Reference** | **PDB Date** |
| --- | --- | --- | --- | --- | --- | --- | --- |
|  |  |  |  |  |  |  |  |
| 5-HT2A | 7VOE | A (Rhodopsin) | Active | Aripirazole | Agonist | [**10.1038/s41593-021-00971-w**](http://dx.doi.org/10.1038/s41593-021-00971-w) | 2021.12.2 |
| 5-HT2A | 7VOD | A (Rhodopsin) | Active | Cariprazine | Agonist | [**10.1038/s41593-021-00971-w**](http://dx.doi.org/10.1038/s41593-021-00971-w) | 2021.12.22 |
| 5-HT1A | 7E2X | A (Rhodopsin) | Active | Apo (no ligand) | Apo (no ligand) | [10.1038/S41586-021-03376-8](https://dx.doi.org/10.1038/S41586-021-03376-8) | 2021.04.14 |
| 5-HT1A | 7E2Z | A (Rhodopsin) | Active | Aripiprazole | Agonist (partial) | [10.1038/S41586-021-03376-8](https://dx.doi.org/10.1038/S41586-021-03376-8) | 2021.04.14 |
| 5-HT1A | 7E2Y | A (Rhodopsin) | Active | Serotonin | Agonist | [10.1038/S41586-021-03376-8](https://dx.doi.org/10.1038/S41586-021-03376-8) | 2021.04.14 |
| 5-HT1D | 7E+32 | A (Rhodopsin) | Active | Serotonin | Agonist | [10.1038/S41586-021-03376-8](https://dx.doi.org/10.1038/S41586-021-03376-8) | 2021.04.21 |
| 5-HT1E | 7E+33 | A (Rhodopsin) | Active | 3-(1-Methylpiperidin-4-yl)-1H-indol-5-ol | Agonist | [10.1038/S41586-021-03376-8](https://dx.doi.org/10.1038/S41586-021-03376-8) | 2021.04.14 |
| 5-HT1F | 7EXD | A (Rhodopsin) | Active | Lasmiditan | Agonist | [10.1038/S41422-021-00527-4](https://dx.doi.org/10.1038/S41422-021-00527-4) | 2021.08.04 |
| 5-HT2A | 6WHA | A (Rhodopsin) | Active | 25-CN-NBOH | Agonist | [10.1016/J.CELL.2020.08.024](https://dx.doi.org/10.1016/J.CELL.2020.08.024) | 2020.09.23 |
| D1 | 7JOZ | A (Rhodopsin) | Active | 6-{4-[(furo[3,2-c]pyridin-4-yl)oxy]-2-methylphenyl}-1,5-dimethylpyrimidine-2,4(1H,3H)-dione | Agonist | [**10.1038/s41467-021-23519-9**](http://dx.doi.org/10.1038/s41467-021-23519-9) | 2021.04.14 |
| D1 | 7JV5 | A (Rhodopsin) | Active | SKF-81297 | Agonist | [10.1016/J.CELL.2021.01.027](https://dx.doi.org/10.1016/J.CELL.2021.01.027) | 2021.02.24 |
| D1 | 7JVQ | A (Rhodopsin) | Active | Apomorphine | Agonist | [10.1016/J.CELL.2021.01.027](https://dx.doi.org/10.1016/J.CELL.2021.01.027) | 2021.02.24 |
| D1 | 7JVP | A (Rhodopsin) | Active | SKF-83959 | Agonist | [10.1016/J.CELL.2021.01.027](https://dx.doi.org/10.1016/J.CELL.2021.01.027) | 2021.02.24 |
| D1 | 7CRH | A (Rhodopsin) | Active | SKF-83959 | Agonist | [10.1016/J.CELL.2021.01.028](https://dx.doi.org/10.1016/J.CELL.2021.01.028) | 2021.03.03 |
| D1 | 7CKX | A (Rhodopsin) | Active | A77636 | Agonist | [10.1016/J.CELL.2021.01.028](https://dx.doi.org/10.1016/J.CELL.2021.01.028) | 2021.03.03 |
| D1 | 7CKW | A (Rhodopsin) | Active | Fenoldopam | Agonist | [10.1016/J.CELL.2021.01.028](https://dx.doi.org/10.1016/J.CELL.2021.01.028) | 2021.03.03 |
| D1 | 7CKY | A (Rhodopsin) | Active | PW0464 | Agonist | [10.1016/J.CELL.2021.01.028](https://dx.doi.org/10.1016/J.CELL.2021.01.028) | 2021.03.03 |
| D1 | 7CKZ | A (Rhodopsin) | Active | dopamine | Agonist | [10.1016/J.CELL.2021.01.028](https://dx.doi.org/10.1016/J.CELL.2021.01.028) | 2021.03.03 |
|  |  |  |  | Mevidalen | PAM |  |  |
| D1 | 7LJC | A (Rhodopsin) | Active | SKF-81297 | Agonist | [10.1101/2021.02.07.430101](https://dx.doi.org/10.1101/2021.02.07.430101) | 2021.03.03 |
|  |  |  |  | Mevidalen | PAM |  |  |
| D1 | 7LJD | A (Rhodopsin) | Active | dopamine | Agonist | [10.1101/2021.02.07.430101](https://dx.doi.org/10.1101/2021.02.07.430101) | 2021.03.03 |
|  |  |  |  | Mevidalen | PAM |  |  |
| D2 | 7JVR | A (Rhodopsin) | Active | Bromocriptine | Agonist | [10.1016/J.CELL.2021.01.027](https://dx.doi.org/10.1016/J.CELL.2021.01.027) | 2021.02.24 |
| D2 | 6VMS | A (Rhodopsin) | Active | Bromocriptine | Agonist | [10.1038/S41586-020-2379-5](https://dx.doi.org/10.1038/S41586-020-2379-5) | 2020.06.17 |
| D3 | 7CMV | A (Rhodopsin) | Active | PD-128907 | Agonist | [10.1016/J.MOLCEL.2021.01.003](https://dx.doi.org/10.1016/J.MOLCEL.2021.01.003) | 2021.03.10 |
| D3 | 7CMU | A (Rhodopsin) | Active | Pramipexole | Agonist | [10.1016/J.MOLCEL.2021.01.003](https://dx.doi.org/10.1016/J.MOLCEL.2021.01.003) | 2021.03.10 |
| H1 | 7DFL | A (Rhodopsin) | Active | Histamine | Agonist | [**10.1038/s41467-021-22427-2**](http://dx.doi.org/10.1038/s41467-021-22427-2) | 2021.03.31 |
| M2 | 6U1N | A (Rhodopsin) | Active | 2CU | Agonist | [10.1038/S41586-020-1954-0](https://dx.doi.org/10.1038/S41586-020-1954-0) | 2020.02.26 |
|  |  |  |  |  |  |  |  |
| β 2B | 6K41 | A (Rhodopsin) | Active | Dexmedetomidine | Agonist | [10.1038/S41589-020-0492-2](https://dx.doi.org/10.1038/S41589-020-0492-2) | 2020.04.15 |
| β 2B | 6K42 | A (Rhodopsin) | Active | Dexmedetomidine | Agonist | [10.1038/S41589-020-0492-2](https://dx.doi.org/10.1038/S41589-020-0492-2) | 2020.04.15 |
| β 1 | 7JJO | A (Rhodopsin) | Active | Isoprenaline | Agonist | [10.1016/J.MOLCEL.2020.08.001](https://dx.doi.org/10.1016/J.MOLCEL.2020.08.001) | 2020.09.02 |
| β 1 | 7BU7 | A (Rhodopsin) | Active | BI-167107 | Agonist | [10.1038/S41422-020-00424-2](https://dx.doi.org/10.1038/S41422-020-00424-2) | 2020.12.02 |
| β 1 | 7BU6 | A (Rhodopsin) | Active | noradrenaline | Agonist | [10.1038/S41422-020-00424-2](https://dx.doi.org/10.1038/S41422-020-00424-2) | 2020.12.02 |
| β 1 | 7BTS | A (Rhodopsin) | Active | Epinephrine | Agonist | [10.1038/S41422-020-00424-2](https://dx.doi.org/10.1038/S41422-020-00424-2) | 2020.12.02 |
| β 1 | 6TKO | A (Rhodopsin) | Active | Formoterol | Agonist | [10.1038/S41586-020-2419-1](https://dx.doi.org/10.1038/S41586-020-2419-1) | 2020.06.17 |
|  |  |  |  |  |  |  |  |
| β 2 | 7BZ2 | A (Rhodopsin) | Active | Formoterol | Agonist | [10.1038/S41421-020-0176-9](https://dx.doi.org/10.1038/S41421-020-0176-9) | 2020.08.05 |
| β 2 | 7DHI | A (Rhodopsin) | Active | Salbutamol | Agonist | [10.1093/NSR/NWAA284](https://dx.doi.org/10.1093/NSR/NWAA284) | 2020.12.16 |
| β 2 | 7DHR | A (Rhodopsin) | Active | Isoprenaline | Agonist | [10.1093/NSR/NWAA284](https://dx.doi.org/10.1093/NSR/NWAA284) | 2020.12.16 |
| β 3 | 7DH5 | A (Rhodopsin) | Active | Mirabegron | Agonist | [10.1016/J.MOLCEL.2021.06.024](https://dx.doi.org/10.1016/J.MOLCEL.2021.06.024) | 2021.08.04 |

# Table S3. D_3_ selective SAR study from PCMPA, bindig affinity data^1^

| **Cmpd** | **Structure** | **Ki (nM)** | | | | | |
| --- | --- | --- | --- | --- | --- | --- | --- |
|  |  | **D_1_R** | **D_2_R** | **D_3_R** | **D_4_R** | **D_5_R** | **5-HT_2C_** |
| **8** |  | *-* | - | 1712 | - | - | 3.4 |
| **9** |  | - | - | 811 | - | - | 4.2 |
| **10** |  | 1918 | 233.0 | 36.7 | 130.0 | >5000 | 535.0 |
| **11** |  | 636 | 216.0 | 23.7 | 163.0 | >5000 | 274.0 |
| **12** |  | 368.7 | 1502.0 | 524.8 | 962.3 | 1079.8 | NT |
| **13**  **BP-897** |  | 1132 | 247.0 | 3.1 | 634.0 | 1223 | 218.0 |
| **14** |  | 967 | 1083.9 | 14.5 | 844 | 2203 | 63.1 |
| **15** |  | 1084 | 380 | 23.4 | 222 | >5000 | 49.0 |
| **16** |  | 871 | 507 | 11.2 | 3760 | 789 | 68.0 |
| **17** |  | 1758 | 3144 | 4.0 | 1142 | >5000 | 53.0 |
| (1*S*,2*S*)-**17a** |  | 1071 | 1230 | 3.8 | 851 | >5000 | 50.1 |
| (1*R*,2*R*)-**17b** |  | 4898 | 1349 | 4.1 | 575 | >5000 | 1122 |
| **18** |  | 891 | 2105 | 2.0 | 1339 | 4018 | 123 |
| (1*S*,2*S*)-**18a** |  | 1047 | 1148 | 20.8 | 776 | >5000 | 138 |
| (1*R*,2*R*)-**18b** |  | 1288 | 676 | 4.4 | 813 | >5000 | 513 |
| **19** |  | 891 | 1502 | 2.6 | 1131 | 3500 | 105 |
| (1*S*,2*S*)-**19a** |  | 1122 | 992 | 12.8 | 676 | >5000 | 61.7 |
| (1*R*,2*R*)-**19b** |  | 1380 | 537 | 2.2 | 1047 | >5000 | 513 |
| **20** |  | 1647 | 2362 | 1.2 | 2011 | >5000 | 97.7 |
| (1*S*,2*S*)-**20a** |  | 2344 | 1023 | 5.3 | 912 | >5000 | 44.7 |
| (1*R*,2*R*)-**20b** |  | 1349 | 550 | 1.5 | 676 | >5000 | 417 |

# Table S4. SAR study from PD-128907 and PF-592379, binding affinity data^2^

| **vs [^3^H]-(R)—(+)-7-OH-DPAT** | | | | | | |
| --- | --- | --- | --- | --- | --- | --- |
| **Cmpd** | **Structure** | **D_2_R K_i_ (nM)** | **D_3_R K_i_ (nM)** | **D_4_R K_i_ (nM)** | **D_2_R/D_3_R** | **D_4_R/D_3_R** |
| **PD-128907** |  | 20.5 | 1.69 | 26.6 | 12.1 | 15.7 |
| **PF-592379**^3^ |  | >10000 | 215 | 4165 | >46,5 | 19,4 |
| **21**  **(3:1)** |  | 1740 | 185 | 292 | 9.41 | 1.58 |
| **22** |  | 7100 | 1520 | ND | 4.67 | ND |
| **23** |  | 2340 | 424 | ND | 5.52 | ND |
| **24** |  | 2600 | 24200 | ND | 0.110 | ND |
| **25** |  | 34.6 | 31.2 | ND | 1.1 | ND |
| **26** |  | 5220 | 6470 | ND | 0.807 | ND |
| **27** |  | 134 | 5.96 | 357 | 22.5 | 59.9 |
| **rac-trans-28** |  | 106 | 2.84 | 315 | 37.3 | 111 |
| **28a** |  | 87.8 | 1.85 | 286 | 47.5 | 155 |
| **28b** |  | 831 | 282 | 2930 | 2.95 | 10.4 |
| **Radioligand Competition Binding Assays Performed on HEK293 Cells Stably Expressing D_2_R, D_3_R, D_1_R, and μORc**^4^ | | | | | | |
| **Cmpd** | **Structure** | **D_2_R K_i_ (nM)** | **D_3_R K_i_ (nM)** | **D_1_R K_i_ (µM)** | **µOR K_i_ (µM)** | |
| **PD-128907** |  | 28 | 16.7 | >100 | 36.6 | |
| **29** |  | 7580 | 5620 | >100 | 77.9 | |
| **30** |  | >10 | >10 | NT | NT | |
| **28a** |  | 87.8 | 1.85 | >100 | 16,9 | |
| **31** |  | 1010 | 423 | >100 | 21.9 | |
| **32** |  | 104 | 21.6 | >100 | 0.739 | |

# Table S5. Binding data for 3-thiophenephenyl and 4-thiazolylphenyl fluoridesubstituted N-phenylpiperazine analogs^5^

|  | | | | | | |
| --- | --- | --- | --- | --- | --- | --- |
| **Cmpd** | **Structure** | | **Ki (nM)** | | **D_2_/D_3_** | **5-HT_1A_ inhib. %** |
|  | **R_1_** | **R_2_** | **D_2_R** | **D_3_R** |  |  |
| **33** | 2-F | - | 384±39.6 | 96.2±16.9 | 4.0 | 20.3±16.2 |
| **34** | 3-F |  | 2516±252 | 1003±118 | 2.5 | 36.1±2.1 |
| **35** | 4-F |  | 3091±269 | 1176±240 | 2.5 | 5.1±11.1 |
| **36** | 2-F, 5-CN |  | 5431±1059 | 726±101 | 7.5 | -18.0±11.1 |
| **37** | 2-F, 4CF_3_ |  | 7522±466 | 1413±59.1 | 5.3 | -11.6±15.4 |
| **38** | 2-OC_2_H_4_F, 4-F |  | 349±21.1 | 351±42.1 | 1.0 | 1.7±21.7 |
| **39** | 2-F |  | 648±100 | 1.4±0.2 | 467 | -3.3±15.1 |
| **40** | 3-F |  | 2334±485 | 7.9±1.4 | 296 | 74.7±1.9 |
| **41** | 4-F |  | 7200±215 | 12.4±1.6 | 579 | -7.7±23.5 |
| **42** | 2-F, 5-CN |  | 4502±565 | 15.5±1.3 | 290 | 2.5±3.5 |
| **43** | 2-F, 4CF_3_ |  | >75000 | 43.3±9.2 | >1800 | 6.1±6.8 |
| **44** | 2-OC_2_H_4_F, 4-F |  | 411±27.3 | 6.1±0.2 | 67.4 | 16.9±24.4 |
| **45** | 2-F |  | 478±71.0 | 2.5±0.22 | 190 | 65.8±5.0 |
| **46** | 3-F |  | 3946±146 | 24.0±5.5 | 165 | 74.1±5.3 |
| **47** | 4-F |  | 4012±694 | 28.6±2.1 | 140 | 12.2±3.1 |
| **48** | 2-F, 5-CN |  | 7717±1497 | 28.1±0.8 | 274 | -5.6±18.3 |
| **49** | 2-F, 4CF_3_ |  | >40000 | 30.9±2.6 | 1390 | 7.6±6.5 |
| **50** | 2-OC_2_H_4_F, 4-F |  | 349±50.3 | 4.8±0.28 | 72.9 | 37.1±24.4 |

References

(1) Tan, L.; Zhou, Q.; Yan, W.; Sun, J.; Kozikowski, A. P.; Zhao, S.; Huang, X.-P.; Cheng, J. Design and Synthesis of Bitopic 2-Phenylcyclopropylmethylamine (PCPMA) Derivatives as Selective Dopamine D3 Receptor Ligands. *J. Med. Chem.* **2020**, *63* (9), 4579–4602. https://doi.org/10.1021/acs.jmedchem.9b01835.

(2) Battiti, F. O.; Cemaj, S. L.; Guerrero, A. M.; Shaik, A. B.; Lam, J.; Rais, R.; Slusher, B. S.; Deschamps, J. R.; Imler, G. H.; Newman, A. H.; Bonifazi, A. The Significance of Chirality in Drug Design and Synthesis of Bitopic Ligands as D _3_ Receptor (D _3_ R) Selective Agonists. *J. Med. Chem.* **2019**, *62* (13), 6287–6314. https://doi.org/10.1021/acs.jmedchem.9b00702.

(3) Collins, G. T.; Butler, P.; Wayman, C.; Ratcliffe, S.; Gupta, P.; Oberhofer, G.; Caine, S. B. Lack of Abuse Potential in a Highly Selective Dopamine D3 Agonist, PF-592,379, in Drug Self-Administration and Drug Discrimination in Rats. *Behavioural Pharmacology* **2012**, *23* (3), 280–291. https://doi.org/10.1097/FBP.0b013e3283536d21.

(4) Battiti, F. O.; Newman, A. H.; Bonifazi, A. Exception That Proves the Rule: Investigation of Privileged Stereochemistry in Designing Dopamine D3R Bitopic Agonists. *ACS Med. Chem. Lett.* **2020**, *11* (10), 1956–1964. https://doi.org/10.1021/acsmedchemlett.9b00660.

(5) Lee, B.; Taylor, M.; Griffin, S. A.; McInnis, T.; Sumien, N.; Mach, R. H.; Luedtke, R. R. Evaluation of Substituted N-Phenylpiperazine Analogs as D3 vs. D2 Dopamine Receptor Subtype Selective Ligands. *Molecules* **2021**, *26* (11), 3182. https://doi.org/10.3390/molecules26113182.
